# Laser Surface Alloying of Austenitic 316L Steel with Boron and Some Metallic Elements: Properties

**DOI:** 10.3390/ma14112987

**Published:** 2021-05-31

**Authors:** Michał Kulka, Daria Mikołajczak, Piotr Dziarski, Dominika Panfil-Pryka

**Affiliations:** 1 Institute of Materials Science and Engineering, Poznan University of Technology, Pl. M.Sklodowskiej-Curie 5, 60-965 Poznan, Poland; piotr.dziarski@put.poznan.pl (P.D.); dominika.panfil-pryka@put.poznan.pl (D.P.-P.); 2 WSK Poznan Ltd., Unii Lubelskiej Street 3, 61-249 Poznan, Poland; daria.mikolajczak02@gmail.com

**Keywords:** laser surface alloying, laser boriding, 316L steel, hardness, wear resistance, corrosion resistance

## Abstract

Austenitic 316L stainless steel is known for its good resistance to corrosion and oxidation. However, under conditions of appreciable mechanical wear, this steel had to demonstrate suitable wear protection. In this study, laser surface alloying with boron and some metallic elements was used in order to improve the hardness and wear behavior of this material. The microstructure was described in the previous paper in detail. The microhardness was measured using Vickers method. The “block-on-ring” technique was used in order to evaluate the wear resistance of laser-alloyed layers, whereas, the potentiodynamic method was applied to evaluate their corrosion behavior. The produced laser-alloyed layers consisted of hard ceramic phases (Fe_2_B, Cr_2_B, Ni_2_B or Ni_3_B borides) in a soft austenitic matrix. The significant increase in hardness and wear resistance was observed in the case of all the laser-alloyed layers in comparison to the untreated 316L steel. The predominant abrasive wear was accompanied by adhesive and oxidative wear evidenced by shallow grooves, adhesion craters and the presence of oxides. The corrosion resistance of laser-alloyed layers was not considerably diminished. The laser-alloyed layer with boron and nickel was the best in this regard, obtaining nearly the same corrosion behavior as the untreated 316L steel.

## 1. Introduction

The main disadvantage of AISI 316L austenitic stainless steel is its relatively low hardness (about 200 HV) which causes the limited use of this material. It would be difficult to harden the austenitic steel using the typical heat treatment, i.e., quenching and tempering, because of the extended stability of an austenitic structure to the room temperature [1]. Therefore, the only way to harden such a steel is via adequate surface treatment in order to produce hard and wear resistant surface layers. It is relatively easy using the physical techniques of surface treatment, especially if the surface is saturated with nitrogen, carbon or boron under glow discharge conditions [2,3,4,5,6,7,8,9,10,11,12,13,14,15,16,17,18,19,20,21,22,23,24,25,26,27,28,29,30,31]. Such techniques are also called plasma or ion processes [32]. In this case, the activation of the surface is carried out during the first step of the process, i.e., sputter cleaning of the surface. This pre-treatment causes removal of the passive layer, consisting of oxides, from the surface. Among these processes, the most important are: low-temperature plasma gas nitriding (LTPGN), high-temperature plasma gas nitriding (HTPGN), low-temperature plasma gas carburizing (LTPGC), low-temperature plasma gas nitrocarburizing (LTPGNC), cathodic plasma electrolytic nitriding (CPEN) or plasma paste boriding (PPB). The conventional thermo-chemical treatment, i.e., boriding [33,34,35,36,37,38,39,40,41,42,43], nitriding [44,45,46,47,48,49] or carburizing [50,51,52] as well as producing the TiN coatings by physical vapor deposition (PVD) [53,54], requires the mechanical or chemical removing these oxides before these processes. It is relatively difficult due to the susceptibility of austenitic steel to re-passivation. The new possibilities, especially in increasing the depth of surface layers produced, appear in the case of laser surface alloying (LSA) [55,56,57,58,59,60,61,62,63]. However, the main problem is how to improve the tribological properties of the austenitic stainless steel without sacrificing its corrosion resistance. 

All the techniques, mentioned above, were applied in order to increase the hardness of the surface layers produced, and, consequently, to improve the wear resistance of the austenitic stainless steels. However, the experimental procedure of wear tests differed, including “block-on-ring” [2,3,31,52,60], “ball-on-disc” [5,15,21,22,23,38,53,62] as well as “pin-on-disc” [8,20,41,57,61] techniques. The tribological properties of the surface layers produced on the 316L steel were evaluated using various measured quantities, such as linear wear [2], volumetric wear [9,19,39,44,45,62], volumetric wear per unit of axial force and friction track (also called specific wear rate [7,20,21,22,35,40,54,61]), mass loss [8,41], relative mass loss [60], mass loss per unit of friction track (also called specific wear rate [53]), coefficient of friction (CoF) [7,8,9,16,37,41,54,57], percentage of the volume removed on carburized samples regarding the noncarburized material [26] or, finally, factor of mass wear intensity, i.e., mass loss per friction surface and unit of friction time during the stabilized wear [31,60]. The measured values, characteristic of the surface layers produced at different treatment parameters, were usually compared to the wear behavior of the untreated austenitic stainless steel.

Many papers, mentioned above, also analyzed the corrosion behavior of the surface layers produced on austenitic steels. Usually, the potentiodynamic method was used for evaluation of their corrosion resistance, and the corrosive medium depended on the predicted applications of the layers. As a consequence of this test, carried out in various corrosive media, the corrosion resistance of the layers was usually evaluated based on the determined polarization curves, which provided important parameters such as corrosion potential (*E_corr_*) and corrosion current density (*I_corr_*). These results were compared to the polarization curve obtained in the case of substrate material, i.e., austenitic steel. Then, the conclusions were formulated regarding the applicability of the proposed surface treatment.

The aim of the present study was to confirm that the laser surface alloying of austenitic 316L steel with boron and selected metallic elements could provide the surface layers of improved tribological behavior without significant sacrificing corrosion resistance of the substrate material. The surface layers were fabricated using alloying materials in the form of powders, which were as follows: boron, boron and Stellite-6, boron and nickel as well as boron and mixture of nickel and chromium. In the previous work [63], only the effects of alloying materials and laser processing parameters (especially laser beam power) on the microstructure and cohesion of laser-alloyed layers were investigated. All the laser-alloyed layers were characterized by a composite microstructure, consisting of hard ceramic phases (Fe_2_B, Cr_2_B, Ni_2_B or Ni_3_B borides) in a soft austenitic matrix. The selected properties of these laser-alloyed layers such as microhardness profiles, wear and corrosion resistance were analyzed in the present work.

## 2. Materials and Methods 

### 2.1. Materials and Specimens

AISI 316L austenitic stainless steel, containing 0.023 wt.% C, 17.45 wt.% Cr, 12.92 wt.% Ni and 2.88 wt.% Mo, was used as the substrate material, i.e., alloyed material subjected to laser surface alloying (LSA). Its chemical composition was shown in the paper [63] in detail according to the data provided by the material supplier. 

The two types of specimens were prepared. The ring-shaped specimens (external diameter 20 mm, internal diameter 12 mm, and height 12 mm) were used in the study of microhardness profiles across the laser-alloyed layers and were also subjected to wear resistance tests. The similar specimens were used in the previous investigation describing the microstructure of the layers produced [63]. They were prepared by machining, and their dimensions were shown in the paper [63] in detail. The second type of specimens was subjected to corrosion tests. In this case, the flat surface of the laser-alloyed specimens was required. Therefore, the samples were prepared in the shape of discs with a diameter of 25 mm and height of 4 mm.

The appropriate powders were applied in order to prepare the alloying materials, including boron and some metallic elements. The amorphous boron B with purity ≥95% and particle size ≤ 1 μm (Sigma Aldrich, Inc., Poznan, Poland) was the main component of the alloying materials. Additionally, the powders of selected metallic elements such as nickel (Ni) powder with purity ≥99.7% and particle size ≤50 μm (Sigma Aldrich, Inc., Poznan, Poland), mixture of nickel and chromium (Ni-Cr) powders with mass ratio 4:1 and particle size ≤25 μm (Euromat, Wroclaw, Poland) as well as Stellite-6 alloy powder with particle size 25–53 μm (Euromat, Wroclaw, Poland) were used in order to prepare the alloying materials. The chemical composition of Stellite-6 powder was specified in the paper [63] in detail. Cobalt was characterized by the highest mass percentage in this powder.

### 2.2. Laser Surface Alloying of 316L Steel

LSA processes of 316L steel could be carried out by the two various techniques: remelting and fusion [32]. In the present study, the remelting technique as the two-stage process, consisting in prior deposition of coating with the alloying material onto the alloyed material (substrate) and subsequent remelting of this coating together with the alloyed material, was used according to the experimental procedure reported by the previous work [63]. 

The powders, including the alloying material mentioned above, were blended with a diluted polyvinyl alcohol solution in order to prepare the paste. The paste was deposited on the outer cylindrical surface of the ring-shaped specimens or the flat surface of discs (made of 316L steel) and subjected to hardness and wear tests or corrosion tests, respectively. All the coatings with alloying materials had a thickness of 200 μm. This thickness was checked by the thickness gauge of coatings Positector 6000 (DeFelsko, Poznan, Poland). This gauge used the phenomenon of the magnetic induction and eddy currents. As in the previous study [63], the four types of alloying material were applied: only amorphous boron, the mixture of amorphous boron and Stellite-6 powders with mass ratio 1:1, the mixture of amorphous boron and nickel powders with mass ratio 1:1, and, finally, the mixture of amorphous boron and Ni-Cr powders with mass ratio 1:1.

The alloying materials were selected intentionally, i.e with premeditation. It was explained in the paper [63] in detail. Based on the paper [60], it was confirmed that LSA with boron only resulted in the formation of hard ceramic phases (iron, chromium and nickel borides) in austenitic matrix. However, such a laser treatment required the relatively high laser beam power (*P*) in order to obtain the dilution ratio (*DR*) of 0.37 or higher [60]. This laser treatment provided the laser-alloyed layers without the microcracks and gas pores. The results of the paper [63] confirmed that the use of alloying materials, composed of boron and such metallic elements as nickel, chromium or cobalt, diminished the laser beam power during LSA, needed to obtain the appropriate dilution ratio which resulted in the formation of the laser-alloyed layers free of microcracks and gas pores. It was caused by the decreased melting points of these elements compared to the melting point of boron. Additionally, the presence of Ni, Cr or Co in alloying material could partially enrich the austenitic matrix by these elements, whereas the formation of nickel, chromium or cobalt borides diminished the concentration of Ni, Cr or Co in austenitic matrix and could cause the worse corrosion resistance of the layers produced.

The second stage of the LSA process was carried out by remelting the prepared coatings together with an alloyed material, i.e., austenitic substrate. Laser processing was performed using a continuous CO_2_ laser TLF 2600 Turbo (TRUMPF, Poznan, Poland). The focusing laser head was coupled with turning lathe in order to make possible its feed motion as well as the rotation of the treated samples. The TEM_01*_ (transverse electromagnetic) multiple mode of the laser beam with a toroidal profile of irradiance was applied in order to achieve the independence of laser treatment effects from the movement direction of the laser beam relative to the laser-alloyed surface. Argon shielding at a pressure of 0.2 MPa provided the appropriate protection of the treated surface against oxidation. 

In the case of ring-shaped specimens, the multiple laser tracks were fabricated along the helical line on the outer cylindrical surface. The two-step technique of LSA by remelting, the equipment used for laser surface alloying of 316L steel as well as and the influence of irradiance profiles on the dimensions of multiple laser tracks were shown and described in the previous paper [63] in detail. The movement of the laser head in relation to the alloyed sample resulted from its rectilinear feed motion and rotation of the sample. The set parameters were as follows: rotational speed of the specimen *n* = 45.85 min^−1^ and feed rate *v_f_* = 0.28 mm per revolution. Hence, the calculated scanning rate *v_l_* = 2.88 m·min^−1^ was a resultant value of tangential speed (*v_t_*) of the specimen and feed rate (*v_f_*). As a consequence of feed rate used, the distance between the axes of adjacent tracks (*f*) was equal to 0.28 mm. The appropriate distance from the bottom edge of a fixing holder of focusing mirror to the treated surface resulted in the laser beam diameter *d* = 2 mm. Based on the values of *d* and *f*, the relatively high overlapping of laser tracks (*O* = 86%) was obtained according to the equation reported by the papers [60,63]. During LSA, the same laser beam powers (*P*) were used as in the previous work [63]. They were as follows: *P* = 1.82 kW for the laser surface alloying with boron only, and *P* = 1.43 kW or *P* = 1.56 kW in the case of the rest alloying materials used, i.e., boron with selected metallic elements. Such values of *P* resulted in the microstructure of laser-alloyed layers which was free of defects, i.e., microcracks and gas pores [63]. 

**Figure 1:**
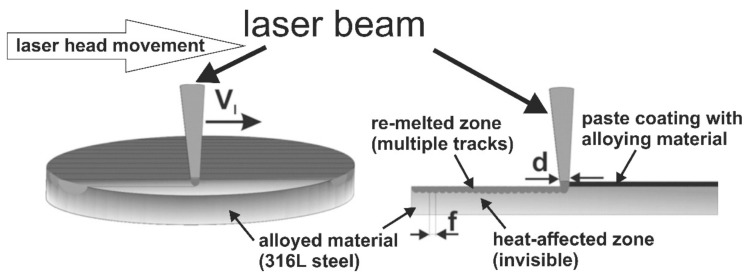
The technique of the multiple laser tracks’ formation along the flat surface of disc-shaped specimens; *d*—laser beam diameter; *v_l_*—scanning rate; *f*—distance from track to track.

The second type of specimens, prepared in the shape of discs and intended for corrosion testing, was subjected to similar laser surface alloying. In this case, the same paste coatings with alloying materials of the thickness of 200 μm were deposited on the flat surfaces of discs during the first stage of LSA. Then, the paste coatings were re-melted together with a substrate material, producing the multiple laser tracks under conditions, which were similar to the laser processing of ring-shaped specimens. First of all, the same laser processing parameters were applied, i.e., laser beam diameter *d* = 2 mm, scanning rate (resulting from the rectilinear movement of laser head) *v_l_* = 2.88 m·min^−1^, and the overlapping (*O*) of 86% (keeping the distance between the axes of adjacent tracks *f* = 0.28 mm). The laser beam power was equal to *P* = 1.82 kW in the case of LSA with boron only and *P* = 1.43 kW in the case of LSA with boron and selected metallic elements. Additionally, in order to keep the similar heating and cooling conditions, the pathway of each simple laser track was equal to the perimeter of the outer surface of ring-shaped specimen, i.e., 2·π·10.1 = 63.46 mm. Each time, the laser head was moved with the distance of 63.46 mm, then returned to the initial position, was moved perpendicularly with the distance *f* = 0.28, and was moved with the distance of 63.46 mm once again. This movement of the laser head enabled the obtainment of the overlapping (*O*) of 86% and lasted until all the surface of disc was treated. The technique of multiple tracks’ formation on the flat surface of disc-shaped specimen was shown in Figure 1. As a consequence, the obtained microstructure was the same as that-produced by LSA in the ring-shaped specimens.

### 2.3. Microhardness Profiles

**Figure 2:**
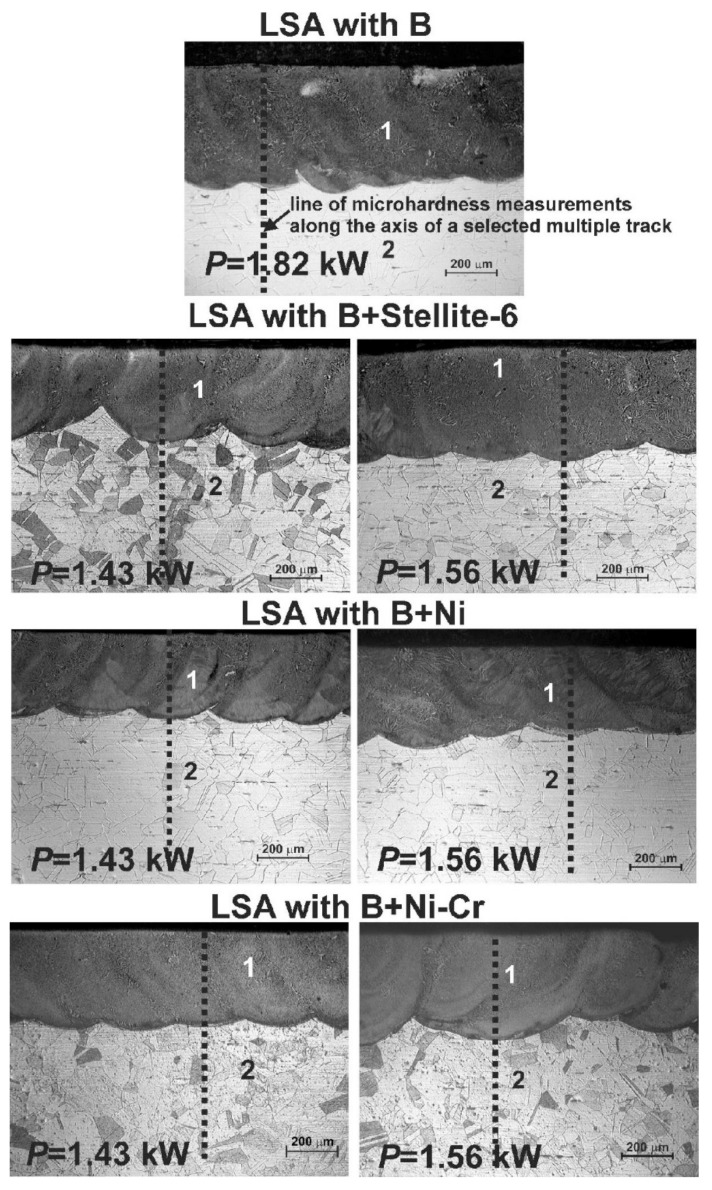
The lines of microhardness measurements along the axis of a selected multiple track based on the microstructure of laser-alloyed layers shown in the paper [63]: 1—re-melted zone (MZ); 2—substrate material (316L steel) without the changes in the microstructure of the heat-affected zone (HAZ).

The microhardness profiles were studied across the laser-alloyed layers using the same metallographic specimens, which were used for microstructure observations and prepared according to the procedure, described in the previous paper [63]. The ring-shaped specimens, cut perpendicularly to the scanning direction, were mounted in a conductive resin, polished and etched using an appropriate reagent in order to reveal microstructure [63]. The microhardness was measured along the axes of the laser tracks by the Vickers method with the use of the 3212 B tester (ZWICK, Poznan, Poland) at the load of 100 gf (about 0.981 N). This procedure enabled to determine the microhardness profiles, showing the microhardness values vs. the distance from the laser-alloyed surface. The lines, along which the microhardness measurements were performed, are shown in Figure 2, presenting the microstructure of laser-alloyed layers [63].

### 2.4. Wear Tests

The wear resistance of the laser-alloyed 316L steel was studied using the same procedure, which was described in the paper [60]. The “block-on-ring” technique, previously often applied after the surface treatment of austenitic steel [2,3,31,52,60], was used in order to characterize the wear behavior of laser-alloyed layers with boron and selected metallic elements. However, the specimens were usually formed in the shape of blocks [2,3,31,52]. The ring-shaped specimens mated with the block-shaped counter-specimens in the paper [60] only. 

**Figure 3:**
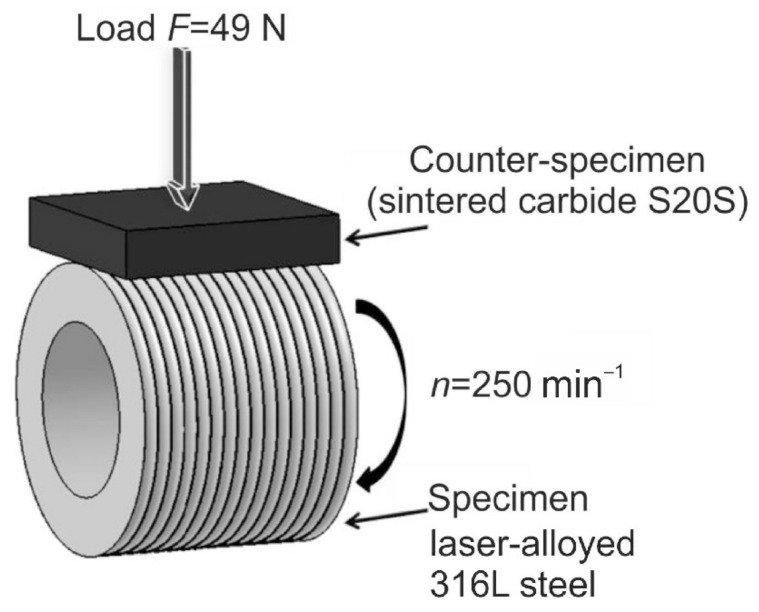
The scheme of the frictional pair during wear tests of laser-alloyed layers, produced in austenitic 316L steel.

In the present study, the same frictional pairs were applied in order to easily compare the wear resistance of the produced laser-alloyed layers to the substrate material (316L steel) as well as laser-alloyed layer, formed with boron only as alloying material [60]. The S20S sintered carbide, consisting of 58 wt.% of WC, 31.5 wt.% of (TiC + TaC +NbC) and 10.5 wt.% of Co as a matrix material, was used as a block-shaped counter-specimen with the dimensions of 12 × 12 × 4 mm. The scheme of wear is shown in Figure 3.

The rotational speed of the laser-alloyed specimen was equal to *n* = 250 min^−1^. It resulted in the tangential specimen speed of 0.26 m·s^−1^ in relation to the fixed counter-specimen that was loaded with a force of 5 kgf, i.e., 49 N. The wear tests were performed under the conditions of dry friction. It was obvious that the surface roughness changed after LSA processes. However, the surfaces of laser-alloyed samples were not specially prepared before the tests. The wear resistance tests lasted 2 h. Every 0.5 h, the position of the counter-specimen was changed this way that the new wear scar was formed on its surface. This method of wear tests was used in order to diminish the adhesive wear, which was observed in the case of wear tests, performed without the change in counter-samples [60]. The analysis of wear resistance was carried out in two ways. The first one consisted in calculations of the factor of mass wear intensity (*I_mw_*) [60]. This factor was defined as the specimen mass loss (Δ*m*) per friction surface (*S*) and unit of time and corresponded to the slope of a straight line in the plot of mass loss per friction surface (Δ*m*/*S*) vs. the friction time (t) [60]. The values of *I_mw_* were calculated using the equation [60]:

(1) $$I_{mw}=\frac{\Delta m}{S\times t}\left(\mathrm{mg}\times {\mathrm{cm}}^{-2}\times h^{-1}\right)$$

where Δ*m* is the mass loss of the specimen (mg), *S* is the friction surface (cm^2^), and *t* is friction time (h).

The outer surface of the laser-alloyed specimen was the friction surface (*S*). Therefore, the external diameter of the sample and its height was measured every 0.5 h of the wear test. The second way of the evaluation of wear resistance took into account the relative mass loss (Δ*m*/*m_i_)* according to the equation [60]:

(2) $$\frac{\Delta m}{m_{i}}=\frac{m_{i}-m_{f}}{m_{i}}$$

where Δ*m* is the mass loss of specimen or counter-specimen (mg), *m_i_* is initial mass of specimen or counter-specimen (mg), *m_f_* is final mass of specimen or counter-specimen (mg).

The assumptions in the wear tests were as follows:

- The first stage of wear consisted in the running-in. During this stage, the relatively high wear of the sample and counter-sample was usually observed,
- The second stage of wear consisted in the steady rate of wear. During this stage, the specimen mass loss (Δ*m*) per friction surface (*S*) obtained the constant value vs. the time of friction. Hence, the wear behavior could be evaluated using the value of mass wear intensity factor (*I_mw_*). The lower *I_mw_* values, the higher wear resistance of the studied material.

In order to identify the mechanisms of wear, the worn surfaces were analyzed using a scanning electron microscope (SEM) equipped with an energy dispersive spectrometer (EDS). The PGT Avalon X-ray microanalyzer (Princeton Gamma Tech, Poznan, Poland) was used. The SEM images could reveal the abrasive or adhesive wear. Hence, the EDS patterns of elements characteristic of the laser-alloyed specimens, i.e., iron, chromium, nickel and molybdenum as well as elements characteristic of the counter-specimen (S20S sintered carbide), i.e., titanium, cobalt and tungsten, were shown on the worn surfaces. Additionally, the EDS patterns of oxygen were analyzed in order to confirm or exclude the oxidative wear.

### 2.5. Corrosion Tests

**Figure 4:**
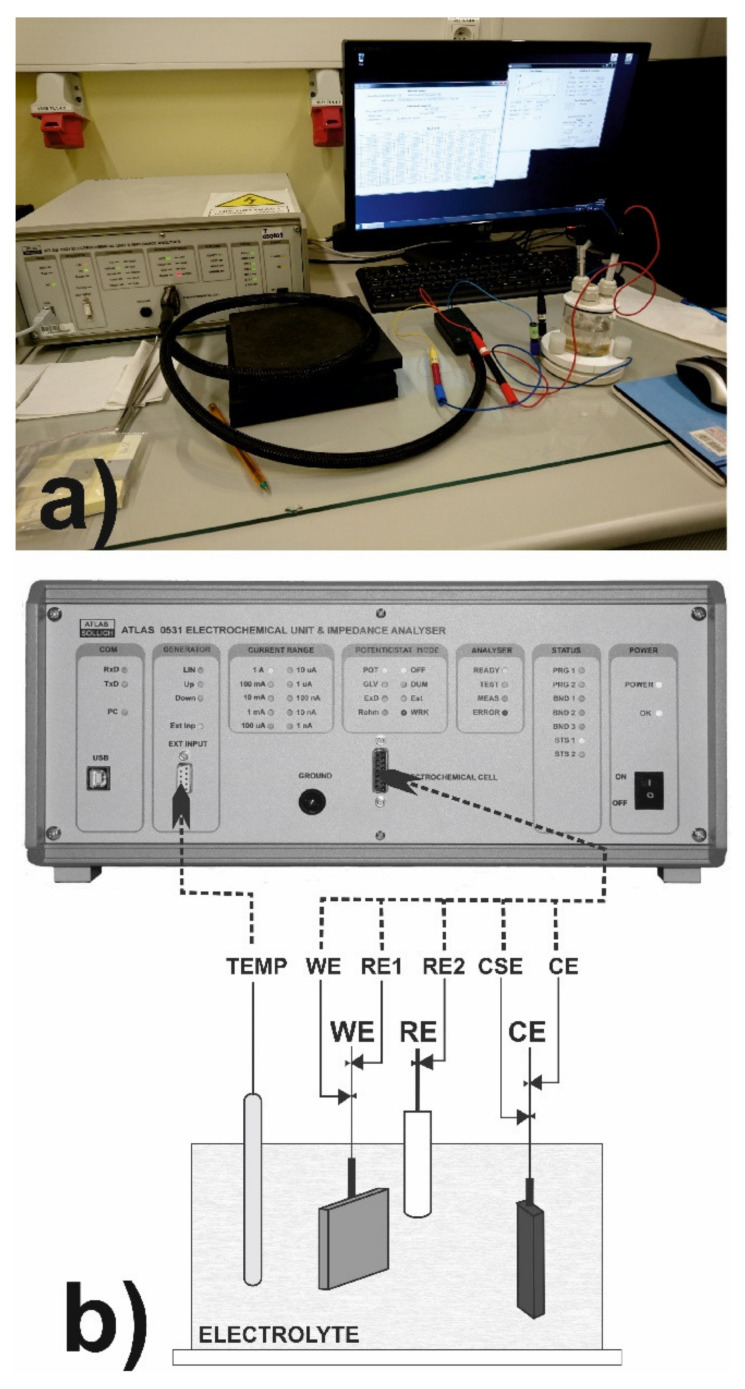
ATLAS 0531 electrochemical unit and impedance analyzer (**a**) and the scheme of 3-terminals cell connections (**b**); WE—working electrode; CE—counter electrode; RE—reference electrode.

The corrosion resistance was investigated using ATLAS 0531 electrochemical unit and impedance analyzer (Figure 4a) in 3.5% NaCl and 1 M H_2_SO_4_ solutions, i.e., the often used solutions in the study of corrosion resistance of austenitic steel as well as the surface layers, produced on this material [9,14,15,16,19,21,22,41,44,52].

This device is a precise three-electrode device which provides measurements of the chrono-volt-amperometric characteristics and impedance spectrums of electrochemical samples. The experiment was arranged as a 1-step potentiodynamic anodic polarization test. During the test, the potential and current were measured by a linear change of forcing potential. The initial value of potential was −2.0 V, whereas the final value of potential was 2.0 V. The rate of potential increasing was equal 0.5 mV·s^−1^. During the test, the three-electrode cell system was used. The scheme of 3-terminals cell connections was presented in Figure 4b. 

In this system the tested sample was a working electrode (WE), the platinum electrode was a counter electrode (CE), and the saturated calomel electrode was a reference electrode (RE). The potential of the working electrode (tested sample) was measured with respect to a counter electrode, which was non-polarizable during the test. The potential (E) and current density (I) of the work electrode were constantly recorded in order to obtain the polarization curve, i.e., E vs. log(I) plot. Based on the polarization curve, the corrosion potential*E_corr_*and corrosion current density*I_corr_*were determined as the two important parameters which connect the fundamental electrochemistry and the practical corrosion behavior of metals. 

The corrosion potential*E_corr_*was defined as an open circuit potential of a working electrode (tested sample).*E_corr_*was a potential at which the rate of anodic dissolution of the working electrode equaled to the rate of cathodic reactions. For this reason, the point on the polarization curve at which the corrosion potential was reached partitioned the plot into the corrosion resistance region and active region, whereas, the corrosion current density*I_corr_*was defined as a dissolution current of the working electrode at the corrosion potential. The value of*I_corr_*was obtained from the intercept of the two linear segments of the Tafel slope of polarization curve.

## 3. Results and Discussion

### 3.1. Microhardness Profiles

**Figure 5:**
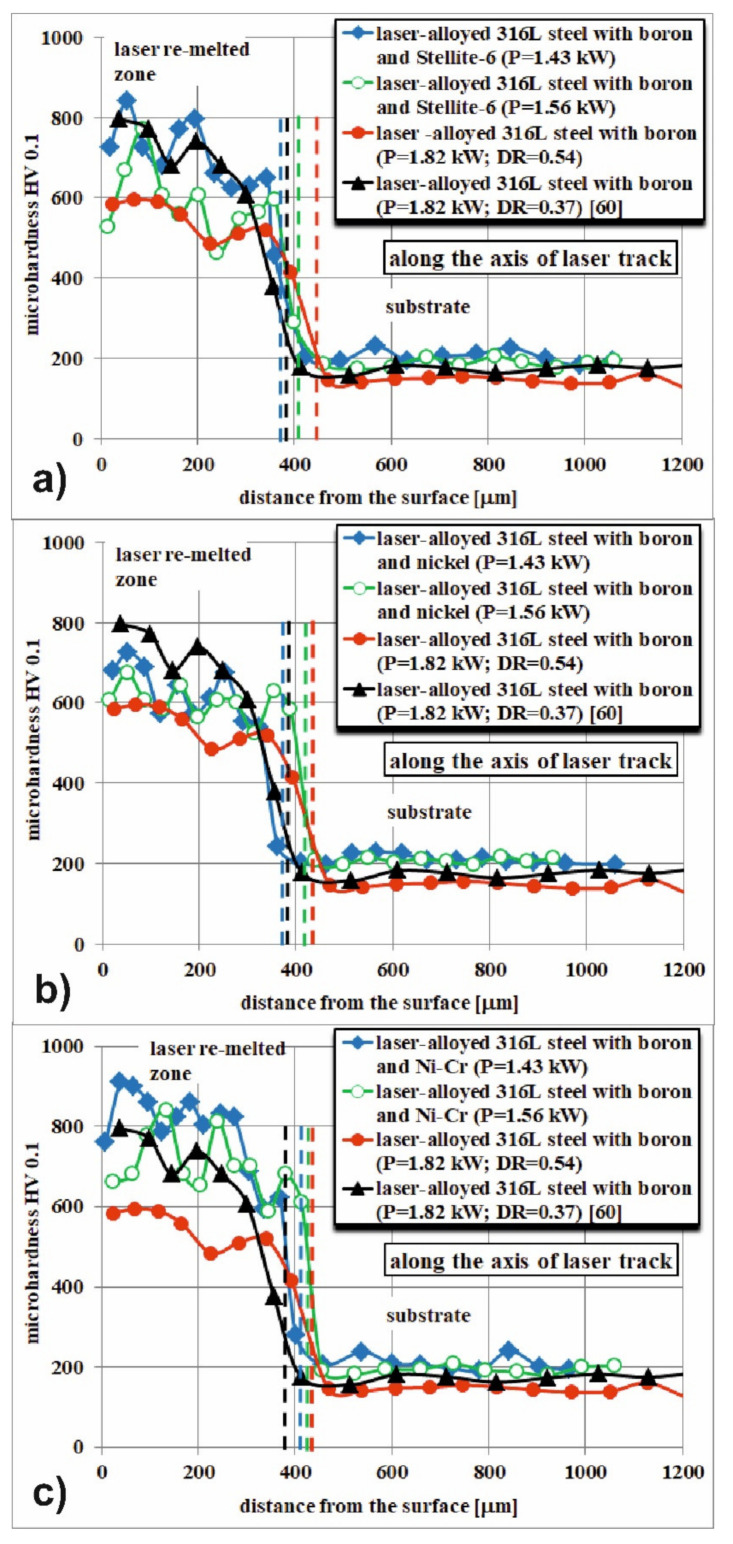
Microhardness profiles along the axes of selected multiple tracks after LSA with boron and Stellite-6 (**a**) with boron and nickel (**b**) and with boron, nickel and chromium (**c**) compared to the LSA with boron only, reported in the present study (*DR* = 0.54) as well as in the paper [60] (*DR* = 0.37).

It was found that the parameters of LSA of 316L austenitic stainless steel, e.g., the type of alloying material and its thickness (*t_C_*), laser beam diameter (*d*), scanning rate (*v_l_*), overlapping (*O*) and laser beam power (*P*) strongly influenced the quality of the produced surface layers [63]. Summarizing the results of the paper [63], it could be concluded that the fabricated laser-alloyed layers were characterized by a composite microstructure, which was composed of the hard ceramic phases (iron, chromium and nickel borides) in a soft austenitic matrix. Due to this composite microstructure, the microhardness indents included both the hard metal borides and the soft austenitic matrix. Hence, the measurements of microhardness usually represented the averaging value of this property, depending on the fraction of hard as well as soft phases, occurring on the penetrated surface. It caused some fluctuations of microhardness in the re-melted zone (MZ), despite the general trend of its diminished value by increasing the distance from the surface. The microhardness profiles along the axes of selected multiple track after LSA with boron and some metallic elements are shown in Figure 5. The results are compared to the laser-alloyed layers with boron only (also called laser-borided layers), fabricated with a dilution ratio (*DR*) of 0.54 during the present study and with *DR* equal to 0.37 [60]. 

Figure 5a shows the microhardness profiles across the two laser-alloyed layers with boron and Stellite-6, produced with the use of laser beam power (*P*) of 1.43 kW and 1.56 kW and resulted in the dilution ratio (*DR*) of 0.41 and 0.48, respectively (according to the data from the paper [63]). The composite microstructure of the re-melted zone (MZ) was composed of the hard iron, chromium and nickel borides (Fe_2_B, Cr_2_B, Ni_2_B) in a soft austenitic matrix (FeCrNiCoCγ phase) [63]. The cobalt borides were not detected. Probably, all the cobalt from the alloying material (Stellite-6 powder) dissolved in the alloyed austenite. The microhardness was measured along the axes of multiple laser tracks, and the determined profiles were compared to the profiles after LSA with boron only, resulting in the dilution ratio of 0.37 [60] and 0.54 (present work). The hardness of the re-melted zone (MZ), i.e., laser-alloyed layer, strongly depended on the type of alloying material and laser processing parameters (in this case—laser beam power used), causing the differences in the dilution ratio and, as a consequence, the various percentages of hard borides in the microstructure. 

The higher hardness close to the surface (about 800 HV) of laser-alloyed layer with boron and Stellite-6 was measured in the case of the use of lower laser beam power (*P* = 1.43 kW). In general, the microhardness profile in laser-alloyed zone (re-melted zone) was in this case comparable to the profile which was obtained after LSA with boron only at a dilution ratio *DR* = 0.37 [60]. Only the depth of MZ was slightly higher because of the higher value of dilution ratio (0.41). However, the composite microstructure of laser-alloyed layer with boron consisted of hard iron, chromium and nickel borides ((Fe_2_B, Cr_2_B, Ni_2_B) and, additionally, M_23_(C,B)_6_ borocarbides in a soft austenitic matrix (FeCrNiCγ phase). The presence of borocarbides could cause the worsened corrosion resistance [60] due to the diminished concentration of chromium in austenitic matrix. Whereas the microhardness profile, obtained at higher laser beam power (*P* = 1.56 kW) was closer to the profile designated for laser boriding resulting in a dilution ratio of 0.54, with a slightly lower depth of re-melted zone due to the lower *DR* value (0.48) and slightly higher hardness in the re-melted zone. The laser-alloyed layer with boron (i.e., laser-borided layer) at *DR* = 0.54 did not contain M_23_(C,B)_6_ borocarbides in MZ. It should advantageously influence its corrosion behavior. In the substrate, microhardness fell to the values which were characteristic of 316L austenitic steel (160–210 HV).

Figure 5b shows the microhardness profiles of laser-alloyed layers with boron and nickel using a laser beam power of 1.43 and 1.56 kW, resulting in a dilution ratio of 0.42 and 0.48, respectively (according to the paper [63]). In the re-melted zone, the presence of a composite microstructure was confirmed. The hard iron, chromium and nickel borides (Fe_2_B, Cr_2_B, Ni_2_B) occurred in a soft FeCrNiCγ phase (austenitic matrix) [63]. The results were compared with microhardness profiles of 316L austenitic steel, laser-alloyed with boron only using the laser beam power of *P* = 1.82 kW at different dilution ratios *DR* (0.37 [60] and 0.54). As in previous cases, the microhardness was measured along the axes of selected multiple laser tracks. The higher values of microhardness close to the surface (approx. 690–740 HV) were measured after LSA with boron and nickel using the lower laser beam power (*P* = 1.43 kW). In the case of laser treatment using a laser beam of higher power (*P* = 1.56 kW), the surface hardness reached 600–680 HV and the depth of the re-melted zone (laser-alloyed zone) increased. In general, the hardness of the re-melted zone was in both cases between the values measured across the laser-alloyed layers with boron only at a dilution ratio (*DR*) of 0.37 [60] and 0.54. They differed slightly in the depth of MZ, resulting from various dilution ratios. The hardness of the substrate below the re-melted zone reached 160–210 HV, i.e., the values characteristic of 316L austenitic steel.

The microhardness profiles of laser-alloyed layers with boron, nickel and chromium at laser beam powers of 1.43 kW and 1.56 kW are shown in Figure 5c. These profiles were compared to the microhardness profiles after laser boriding (LSA with boron exclusively) at various dilution ratios *DR* = 0.37 [60] and *DR* = 0.54 (present work). The composite microstructure of laser-alloyed layers with B, Ni and Cr also consisted of hard iron, chromium and nickel borides (Fe_2_B, Cr_2_B, Ni_2_B) in a soft FeCrNiCγ matrix [63]. Probably, the percentage of chromium borides was increased in this case, resulting in the relatively high hardness of re-melted zone. The higher microhardness of the MZ, up to 940 HV, was obtained in the case of laser-alloyed layer with boron, nickel and chromium using a 1.43 kW laser beam power, resulting in a dilution ratio of 0.43. This was probably due to the appearance in the microstructure of a greater percentage of very hard chromium borides. Slightly lower hardness (670–850 HV) was characteristic of the laser-alloyed layer with boron, nickel and chromium at laser beam of higher power (*P* = 1.56 kW) and a dilution ratio of 0.49. In this case, the obtained hardness of the re-melted zone was comparable to that of the laser-borided layer with a dilution ratio *DR* = 0.37 [60]. Both laser-alloyed layers with boron, nickel and chromium had a microhardness higher than the laser-borided layer with a dilution ratio of 0.54. In the substrate, the hardness was typical of 316L austenitic stainless steel (160–210 HV).

It was quite easy to compare the hardness of the surface layers, produced in the austenitic steel using the various techniques. The maximal hardnesses and averaging depths of the surface layers, fabricated using physical as well as conventional thermochemical techniques, were specified in the Table 1 and Table 2, respectively. The hardness was usually measured using the Vickers method at the various loads both in the case of physical (see Table 1) and thermochemical (see Table 2) techniques used. The Knoop method was rarely applied [10,35]. The hardness was reported more often in GPa [19,26,31,37,38,39,42,49,53,54]. In such cases, the hardness was measured using a nanoindenter with a Berkovich diamond tip [19,26,31,37,39,49,53] or with Vickers diamond tip [54] as well as Vickers microindenter [38,42]. Sometimes Vickers hardness (HV_IT_) was calculated based on the indentation hardness (H_IT_), expressed in GPa and measured by nanoindenter [40].

The various physical techniques of surface treatment of austenitic steel were compared in Table 1, taking into account the maximal hardness and averaging depths of the produced surface layers. The maximal hardness of laser-alloyed layers with boron (595–796 HV0.1) or with boron and selected metallic elements (675–911 HV0.1) ([60] and this work) was relatively low in comparison with the surface layers, produced on the austenitic stainless steel using other physical techniques such as LTPGN processes (572–2175 HV or 720–1100 HK at different loads) [2,3,5,7,8,9,10,23], LTPGNC process (962 HV) [7], HTPGN processes (1060–1340 HV) [3,5,14,15], LTPGC process (11–11.8 GPa) [26] or PPB process (28.093 GPa) [31]. Many hybrid treatments with the use of plasma processes also resulted in higher hardness of the fabricated surface layers, e.g., shot peening (SP) followed by LTPGN or sequential LTPGC and LTPGN (1615–1662 HV or 7.5–11.5 GPa, respectively) [17,19], cold spraying (CS) of 316L steel followed by LTPGN, LTPGC and LTPGNC processes or their various combinations (800–1350 HV) [21], LTPGN process followed by a multi-arc ion plating (MAIP) (2280 HV) [23] as well as TiN coatings produced by PVD technique (18.7–26 GPa) [23,24]. Only some of the surface layers, produced using the plasma processes, were characterized by comparable or lower hardness, e.g., the layer fabricated using LTPGN (5–9 GPa) [19], LTPGC (570–930 HV) [27,28] or CPEN (438 HV) [16]. The previously used laser surface alloying of austenitic steel [57,59] resulted in diminished hardness (410–480 HV) in comparison with the LSA processes presented in this study. However, the laser-alloyed layers with boron as well as with boron and selected metallic elements obtained significantly higher depths which were in the range of 338–432 μm (see Table 1). Only the LSA with NiCoCrB powder [59] resulted in comparable depth of the produced surface layer (260–740 μm). The depths of hardened surface layers, fabricated using the plasma or PVD techniques, ranged from 1.4 μm [53] to 90 μm [19] and were much thinner. 

The maximal hardness values and averaging depths of the surface layers measured after the surface treatment of austenitic steel using various thermochemical techniques were specified in Table 2. All the typical thermochemical processes resulted in a hardness, which was significantly higher than that-measured after laser surface alloying presented in this study, i.e., LSA with boron as well as with boron and selected metallic elements. The extremely high hardness of the surface layers, produced on austenitic steel, was obtained after powder-pack boriding (P-PB). The borided layers were characterized by a maximal hardness in the range of: 1580–2000 HV [33,40,41], 1836–2227 HK [35] or 18–24 GPa [37,38,39]. The comparable hardness (22 GPa) was measured after boriding in liquid medium [42]. Surface mechanical attrition treatment (SMAT) followed by P-PB process resulted in a hardness of about 2000 HV [34], whereas the diffusion annealing process after P-PB provided the borided layer with a diminished hardness (15 GPa) due to the elimination of the hard FeB phase from the microstructure [37]. The hybrid surface layers, produced by powder-pack boriding (P-PB) followed by powder-pack chromizing (P-PCr), were characterized by a maximal hardness of 1800 HV [41]. The same processes, carried out in reverse order (P-PCr + P-PB), resulted in a formation of the layers with a maximal hardness of 1610 HV [41]. The hardness of nitride layers was slightly higher than-those produced using LSA. LTGN (low-temperature gas nitriding) provided the surface layer of maximal hardness of 1300 HV [44]. The same process, carried out after HVOF-spraying of 316L steel [45], provided the hardness in the range of 874–1005 HV. The comparable maximal hardness (11.5 GPa) was obtained after nitriding in liquid medium [49]. Low-temperature carburizing (LTC) [52] resulted in a maximal hardness of 1100 HV. However, the averaging depths (1.8–87 μm) of the surface layers, fabricated using the typical thermochemical techniques, were considerably lower in comparison with the laser-alloyed layers (338–432 μm).

Summarizing, although the hardness of laser-alloyed layers with boron as well as with boron and selected metallic elements was lower, their averaging depths were significantly higher than the depths of the surface layers produced on austenitic stainless steels using other physical techniques and thermochemical treatments (see Table 1 and Table 2). These relatively high depths of the hardened surface layers could be important under conditions of appreciable mechanical wear.

### 3.2. Wear Resistance

**Figure 6:**
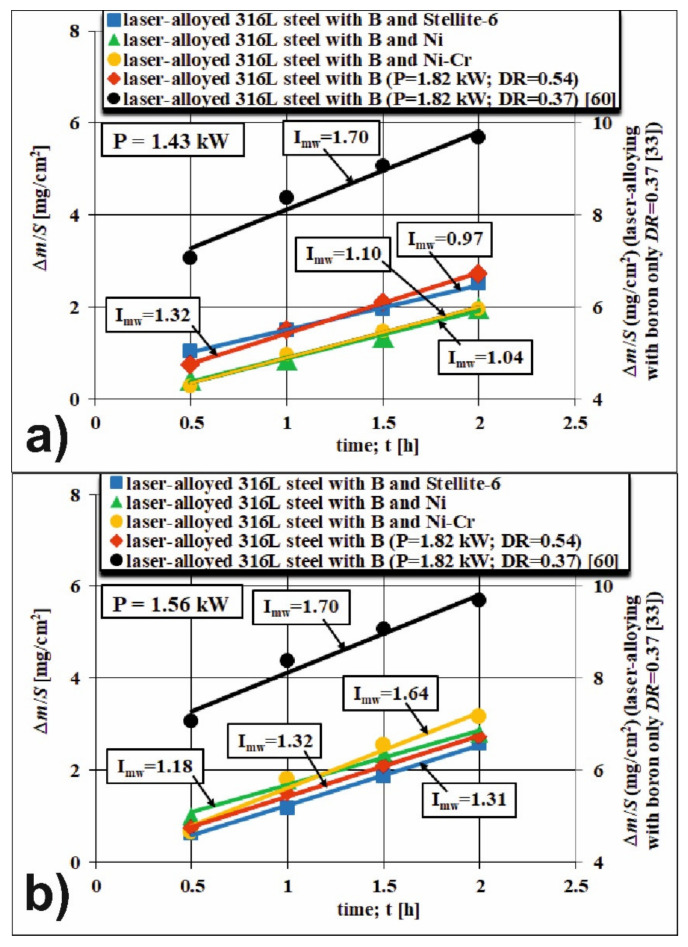
Results of wear tests of laser-alloyed layers with boron and selected metallic elements using the laser beam power *P* = 1.43 kW (**a**) and *P* = 1.56 kW (**b**) compared to the laser-alloyed layers with boron only at *P* = 1.82 kW, reported in the present study (*DR* = 0.54) as well as in the paper [60] (*DR* = 0.37); the mass loss per a unit of friction surface vs. time of friction after the two-hour wear test with the change of counter-specimen at every 0.5 h.

**Figure 7:**
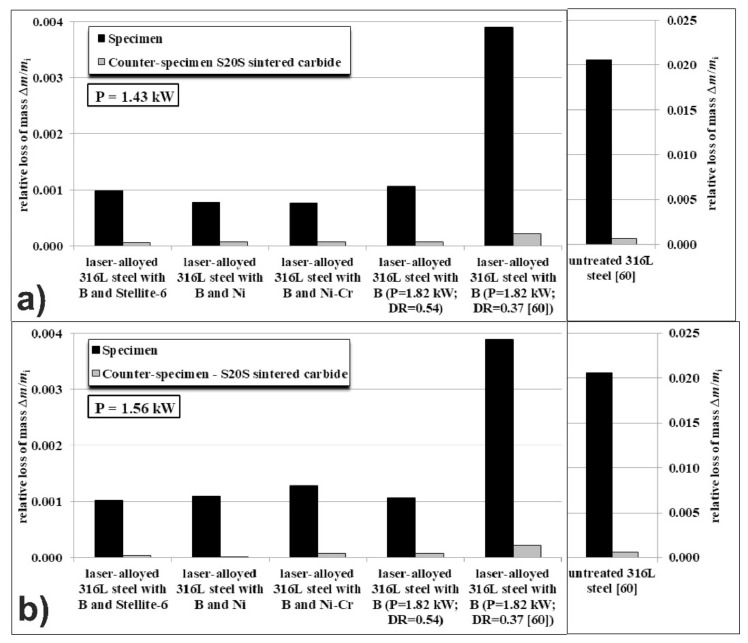
Results of wear tests of laser-alloyed layers with boron and selected metallic elements using the laser beam power *P* = 1.43 kW (**a**) and *P* = 1.56 kW (**b**) compared to the laser-alloyed layers with boron only at *P* = 1.82 kW, reported in the present study (*DR* = 0.54) as well as in the paper [60] (*DR* = 0.37); the relative mass loss of specimens and counter-specimens after the two-hour wear test with the change of counter-specimen at every 0.5 h.

The wear resistance was studied for all the produced laser-alloyed layers with boron and with boron and selected metallic elements. Their wear behavior was investigated for 2 h with a change in the counter-specimen every 0.5 h. The results are presented in Figure 6 and Figure 7 and are compared to the laser-alloyed layer with boron only, reported in the previous study [60]. The mass loss was measured every half hour and the measurement result was divided by the sample surface (friction surface, *S*). This way calculated values were shown vs. the time of friction in order to determine the mass wear intensity factors. The mass wear intensity factor (*I_mw_*) is defined as a mass loss of the specimen per unit of friction surface during a unit of time. It corresponds to the slope of a straight line in the Δ*m*/*S*-time (*t*) coordinate system and represents the wear behavior during the mid-age period when a steady rate of wear occurs (Figure 6). Simultaneously, the values of relative mass loss Δ*m*/*m_i_* of laser-alloyed specimens as well as counter-specimens (made of S20S sintered carbide) were measured and compared (Figure 7).

Based on the previous study [60], the untreated 316L austenitic stainless steel was characterized by a relatively high mass wear intensity factor *I_mw_* = 26.12 mg·cm^−2^·h^−1^. In the case of all the laser-alloyed layers, the values of *I_mw_* were significantly lower (Figure 6). In general, the wear of the 316L steel, subjected to LSA processes, was approximately 15–26-times lower. The mass wear intensity factor, measured during the wear test of laser-alloyed layer with boron at a dilution ratio *DR* = 0.37 [60] was approximately 15-times lower, obtaining the value of 1.70 mg·cm^−2^·h^−1^. The similar layer, produced in the present study at higher dilution ratio (0.54), was characterized by *I_mw_* value of 1.32 mg·cm^−2^·h^−1^, which was approximately 20-times lower compared to the factor of untreated 316L steel. The reduction in *I_mw_* value was achieved despite the lower hardness of this surface layer. The LSA process of 316L steel with boron only required the relatively high laser beam power (*P* = 1.82 kW) in order to produce the surface layers without such defects as microcracks or gas pores. Summarizing, the evaluation of the wear behavior using mass wear intensity factors indicated the considerable increase in wear resistance of laser-alloyed layers with boron in comparison with untreated 316L steel.

The laser-alloyed layers with boron and Stellite-6 (B and Stellite-6) were fabricated on the surface of 316L steel using the two various laser beam powers: *P* = 1.43 kW and *P* = 1.56 kW. It was found that the use of alloying material, consisting of amorphous boron and Stellite-6 powders, allowed the obtainment of a surface layer without defects in the form of micro-cracks or gas pores with lower power of the laser beam [63]. The lower melting point of Stellite-6 powder (1285 °C) compared to boron (2076 °C) made the alloying material easier to melt with the substrate (316L steel) than the alloying material consisting only of boron. Hence, the laser beam power could be lower than-that applied during laser alloying with boron only. Simultaneously, at the mass ratio of boron to Stellite-6 equal to 1:1, the volume percentage of boron (about 78.3 vol%) predominated, taking into account its considerably lower density (2.34 g·cm^−3^) compared to Stellite-6 powder (8.44 g·cm^−3^). The wear resistance was analyzed based on the values of mass wear intensity factors in the mid-age period, when the steady rate of wear occurs. These values after two-hour wear tests with a change in the counter-specimen every 0.5 h were calculated for the two used laser beam powers: *P* = 1.43 kW (Figure 6a) and *P* = 1.56 kW (Figure 6b). The results of wear tests provided the lower mass wear intensity factors (*I_mw_* = 0.97 mg·cm^−2^·h^−1^ and *I_mw_* = 1.31 mg·cm^−2^·h^−1^, respectively) in comparison with the both laser-alloyed layers with boron only. When compared to the untreated 316L steel, the calculated values of *I_mw_* were about 26-times and 20-times lower, respectively. The diminished hardness of the remelted zone, produced at higher laser beam power with the increased dilution ratio (0.48) [63], could be the reason for the higher *I_mw_* value.

The relatively low melting point of nickel (1455 °C) compared to boron (2076 °C) also facilitated the remelting of the alloying material together with the substrate (316L steel). Hence, as in the previous case, the use of the modified laser alloying material (a mixture of boron and nickel powders) allowed obtainment of the layer without the defects such as micro-cracks or gas pores using a diminished laser beam power [63]. Therefore, the laser-alloyed layers with boron and nickel (B and Ni) were produced using the same values of laser beam power (*P* = 1.43 kW and *P* = 1.56 kW) as the laser-alloyed layers with B and Stellite-6. The mass ratio of boron to nickel powder was equal to 1:1. This meant that the volume percentage of boron in the alloying material (about 79.2 vol%) still predominated, taking into account its considerably lower density (2.34 g·cm^−3^) compared to nickel powder (8.908 g·cm^−3^). The values of mass wear intensity factors were analyzed in the mid-age period for the samples which were laser-alloyed using the two used laser beam powers: *P* = 1.43 kW (Figure 6a) and *P* = 1.56 kW (Figure 6b). The calculated values of *I_mw_* (1.04 mg·cm^−2^·h^−1^ and 1.18 mg·cm^−2^·h^−1^, respectively) were approximately 25-times and 22-times lower in comparison with the untreated 316L steel and considerably lower than the values characteristic of the laser-alloyed layers solely with boron, reported in the previous study at *DR* = 0.37 (1.70 mg·cm^−2^·h^−1^) [60] as well as in the present work using *DR* = 0.54 (1.32 mg·cm^−2^·h^−1^). Probably, the lower hardness of the re-melted zone (due to the higher dilution ratio *DR* = 0.48 [63]), was the reason for the increased *I_mw_* value for the laser-alloyed layer which was formed at higher laser beam power (*P* = 1.56 kW).

The use of an alloying material, consisting of boron and Ni-Cr powders, also facilitated its remelting with the substrate material (316L steel) due to the low melting point of nickel (1455 °C) as well as chromium (1857 °C) in comparison with boron (2076 °C). Hence, this modified alloying material resulted in the formation of the laser-alloyed layers without microcracks and gas pores using the diminished laser beam power (*P* = 1.43 kW or *P* = 1.56 kW) [63]. It should be noted that the ratio of nickel to chromium in Ni-Cr powder was equal to 4:1. Therefore, the percentage of nickel was predominating. The mass ratio of the constituents of the alloying material (B:Ni-Cr) was equal to 1:1 and meant that the volume percentage of boron was still predominant (approximately 78.5%) due to its much lower density (2.34 g·cm^−3^) compared to nickel powder (8908 g·cm^−3^) or chromium powder (7.14 g·cm^−3^). Similar to the previous cases, the laser-alloyed austenitic 316L steel with boron and Ni-Cr was subjected to two-hour wear tests with a change in the counter-specimen every 0.5 h. The measured Δ*m*/*S* values vs. time of friction (*t*) are shown in Figure 6a,b for the laser-alloyed layers produced using the laser beam power of 1.43 kW and 1.56 kW, respectively. The calculated values of mass wear intensity factors during the steady rate of wear were equal to 1.10 and 1.64 mg·cm^−2^·h^−1^, respectively. Hence, the values of *I_mw_* were approximately 24-times and 16-times diminished in comparison with the untreated 316L steel. In the case of laser-alloyed layer, produced using the lower laser beam power (1.43 kW), the value of *I_mw_* was considerably lower in comparison with the both laser-alloyed layers with boron only. However, the use of laser beam power of 1.56 kW resulted in *I_mw_* value which was only slightly lower in comparison with the laser-alloyed layer with boron, produced with a dilution ratio of 0.37 [60]. The diminished hardness of re-melted zone due to the higher dilution ratio (*DR* = 0.49 [63]) could be the probable reason for the increased mass wear intensity factor of the laser-alloyed layer, fabricated using the laser beam power of 1.56 kW. However, this hardness was still relatively high compared to other laser-alloyed layers analyzed. A thorough analysis of the wear process (Figure 6b) showed that the running-in period was probably longer than in previous cases. If the test period from 1 to 2 h was taken into account for the calculation, the value of *I_mw_* would be equal to 1.36 mg·cm^−2^·h^−1^, being already closer to the mass wear intensity factors of other laser-alloyed layers.

Summarizing, the addition of selected metallic elements to the alloying material usually resulted in the reduction in the mass wear intensity factor *I_mw_*, especially, if the laser beam power was lower (1.43 kW). This could be understandable, taking into consideration the higher hardness of the re-melted zone in such a case, resulting in a smaller dilution ratio. However, there was no simple relationship between the hardness of the re-melted zone and the value of *I_mw_*. Some laser-alloyed layers with a relatively high hardness, e.g., laser-alloyed layer with boron only (produced using *DR* = 0.37) or both laser-alloyed layers with boron and Ni-Cr, were not at all characterized by the highest resistance to wear, that is, the lowest mass wear intensity factors. To explain these dependencies, the wear mechanisms of these layers should be analyzed. They will be described later. Based on the presented results, the highest wear resistance, i.e., the lowest *I_mw_* values, was obtained in the case of laser-alloyed layer with boron and Stellite-6 and laser-alloyed layer with boron and nickel, produced using the laser beam power of 1.43 kW.

The second method of wear resistance evaluation consisted in the measurements of relative mass loss Δ*m*/*m_i_* of laser-alloyed specimens as well as counter-specimens (made of S20S sintered carbide). The results are shown in Figure 7 for all the laser-alloyed layers compared to the untreated austenitic 316L steel. They indicated the significant increase in wear resistance of laser-borided layers (laser-alloyed layers with boron) in comparison with untreated 316L steel. Based on the previous study [60], the value of Δ*m*/*m_i_* of the laser-alloyed layer with boron, produced using a dilution ratio of 0.37, was equal to 0.0039 and was approximately 5-times lower than the relative mass loss of the untreated 316L steel (0.0206). An even greater decrease in the relative mass loss was observed for a laser-alloyed layer with boron, which was produced in the present study and was characterized by a dilution ratio of 0.54. In this case, the value of Δ*m*/*m_i_* = 0.0011 was obtained. This meant a 19-fold reduction in relative mass loss compared to 316L steel without surface layer. Simultaneously, the relative mass loss of the counter-specimens (made of S20S sintered carbide) was equal to approximately 0.000217 and 0.000072 in the case of the mating parts made of laser-borided specimens at *DR* = 0.37 [60] or *DR* = 0.54, respectively. Such values were considerably smaller than that of the counter-specimen, which mated with the untreated 316L steel (0.000651) [60].

The relative mass losses of the laser-alloyed specimens with boron and selected metallic elements using the laser beam power 1.43 kW and 1.56 kW as well as corresponding counter-specimens are shown in Figure 7a,b, respectively. The Δ*m*/*m_i_* values of the laser-alloyed specimens with boron and Stellite-6 (B and Stellite-6) as well as of their counter-specimens were slightly smaller than those-obtained for laser-borided 316L steel with a dilution ratio of 0.54 and significantly smaller than those-characteristic of the second laser-borided sample (DR = 0,37) [60]. The relative mass loss of the laser-alloyed specimens was equal to about 0.0010 regardless of the laser beam power used. This meant an approximately 21-fold reduction in relative mass loss compared to the untreated 316L steel. In the case of the counter-specimens, their Δ*m*/*m_i_* values ranged from 0.000036 to 0.000065, depending on the laser beam power used in producing the specimens (1.56 kW and 1.43 kW, respectively). These values were considerably lower in comparison with 316L steel without the surface layer (0.000651) [60].

The relative mass loss of the sample, laser-alloyed with boron and nickel (B and Ni) using laser beam power of 1.43 kW (Figure 7a), was considerably lower compared to the values obtained for the laser-borided 316L steel with a dilution ratio of 0.37 [60] or 0.54. To some extent, this was influenced by a smaller mass loss during the running-in stage (Figure 7a). The value of Δ*m*/*m_i_* was equal to about 0.0008, and this meant an approximately 26-fold reduction in relative mass loss compared to the untreated 316L steel (0.0206) [60]. The counter-specimen, which mated with laser-alloyed specimen, had a similar Δ*m*/*m_i_* value (0.000078) to the counter-specimen that mated with a laser-borided layer with a dilution ratio of 0.54 (0.000072). In the case of the laser-alloyed specimen using the higher laser beam power (1.56 kW), its relative mass loss was significantly lower compared to a laser-borided sample with a dilution ratio of 0.37 [60] and comparable to the value measured for a laser-borided sample with a dilution ratio of 0.54 (Figure 7b). This resulted from the slightly higher mass loss of the considered sample during the running-in stage. The obtained value of Δ*m*/*m_i_* (0.0011) was approximately 19-times diminished in comparison with the untreated 316L steel. The relative mass loss of the counter-specimen (0.000016) was exceptionally low and considerably lower than the Δ*m*/*m_i_* values characteristic of the counter-specimens that mated with the both laser-borided specimens with a dilution ratio of 0.37 and 0.54.

Using the same way, the relative mass losses of laser-alloyed specimens with boron, nickel and chromium (B and Ni-Cr) and their counter-specimens were analyzed. The specimen, produced by laser surface alloying using laser beam power of 1.43 kW, was characterized by Δ*m*/*m_i_* value (0.0008) that was significantly lower than those of laser-borided specimens with a dilution ratio of 0.37 [60] or 0.54. The relatively low mass loss during the running-in stage could cause such a situation. This meant an approximately 26-fold reduction in relative mass loss compared to the untreated 316L steel (0.0206) [60]. In the case of the specimen, which was fabricated using higher laser beam power (1.56 kW), the relative mass loss (0.0013) was significantly lower compared to the laser-borided specimen with a dilution ratio of 0.37 [60] and slightly higher than the value measured for laser-borided specimen with *DR* = 0.54. Undoubtedly, this may have been influenced by a slightly longer duration of the running-in stage. Hence, the relative mass loss of this specimen was approximately 16-times lower than that of the 316L steel without surface layer. The relative mass losses of the counter-specimens, which mated with the laser-alloyed specimens using the laser beam powers of 1.43 and 1.56 kW, were equal to 0.000072 and 0.000075, respectively. These values were comparable to the relative mass loss of the counter-specimen, which mated with the laser-borided specimen with a dilution ratio of 0.54.

Summarizing, the lowest relative mass losses of the specimens were obtained in the case of the treatment, consisting in laser surface alloying with boron and nickel (B and Ni) as well as with boron, nickel and chromium (B and Ni-Cr) using the laser beam power of 1.43 kW. This indicated the high wear resistance of these layers. In the case of laser-alloyed specimen with boron and nickel, its high resistance to wear was also confirmed by the relatively low value of mass wear intensity factor *I_mw_* (Figure 6a).

The different techniques and methods of wear evaluation were reported in literature data regarding the wear behavior of the surface layers produced in austenitic steels. The samples, subjected to LTPGN process, were tested using the “block-on-ring” technique with AISI 52100 steel as a ring-shaped counter-sample [2,3], “ball-on-disc” method using AISI 52100 steel [5,9] or Al_2_O_3_ [7] in the shape of ball as a counter-sample or “pin-on-disc” technique using AISI 1045 steel as a counter-sample in the shape of pin [8]. Unfortunately, in the case of very interesting technique of LTPGN using an active screen [10,11,12,13] the wear resistance of the layers was not studied. The effects of HTPGN process on the tribological properties of austenitic steel were tested using “block-on-ring” [3] or “ball-on-disc” [5,15] techniques using the counter-specimen made of AISI 52100 steel in the shape of ring [3] or ball [5] as well as sapphire ball [15], respectively. After the similar process, reported in the paper [14], the wear resistance was not investigated. The wear tests after cathodic plasma electrolytic nitriding (CPEN) were carried out using the “ball-on-disc” technique with a ball composed of Al_2_O_3_ [16]. The similar wear tests were used after hybrid treatment using the plasma nitriding [7,13,17,18,19,20,21,22,23,24]. The wear behavior after simultaneous low-temperature plasma gas nitrocarburizing (LTPGNC) [7] was studied by the same “ball-on-disc” method with the use of Al_2_O_3_ ball as a counter-sample. The wear resistance of the layer, produced by deposition of Au coating followed by LTPGN using active screen was not provided [13]. The shot peening (SP) process was often used before LTPGN [17,18] or sequential LTPGC (low-temperature plasma gas carburizing) and LTPGN processes [19]. However, the study of wear behavior was provided only in the paper [19] in which the “ball-on-disc’ tests using WC-Co balls were performed. In the paper [20], the laser powder bed fusion (L-PBF), one of the selective laser melting (SLM) methods, was used to create untextured and microtextured surfaces and samples. The samples, fabricated using the 316L steel powder, were tested by the “pin-on-disc” technique with the use of Al_2_O_3_ as a counterpart. Al_2_O_3_ ball was applied as a counter-sample during the wear tests of cold-sprayed 316L powder and LTPGN or low-temperature plasma gas carburizing (LTPGC), cold spraying (CS) of such a powder followed by LTPGC and LTPGN or followed by a simultaneous low-temperature plasma gas nitrocarburizing (LTPGNC) process [21]. The same technique of wear test was used in the case of laser metal deposition (LMD) of 316L steel and nickel powders on the surface of AISI 304 austenitic steel prior to LTPGN [22]. The plasma gas-nitrided layers followed by a multi-arc ion plating (MAIP), producing the WCrTiAlN coatings [23], were tested using “ball-on-disc” method with the GCr15 bearing steel balls as counter-specimens. The wear behavior of physical vapor deposited (PVD) (CrWAlTiSi)N multilayer coating on plasma gas-nitrided AISI 316L steel was not studied [24]. The influence of LTPGC process on the wear resistance of the layers produced was studied using “ball-on-disc” technique with alumina (Al_2_O_3_) ball as a counter-sample [26] or by tribocorrosion test integrating the similar “ball-on-disc” tester with an electrochemical potentiostat [27]. Whereas the papers [25,28] did not study the wear behavior of low-temperature gas carburized layers. The plasma gas borided layer became the interlayer between the austenitic substrate and the thin nanostructured diamond film [29], and its wear resistance was not investigated. Only the microstructures and growth kinetics of the plasma paste borided (PPB) layers were analyzed in the paper [30]. The advantageous influence of the PPB process on the wear resistance of austenitic steel was confirmed using “block-on-ring” technique in which a ring-shaped counter-specimen, made of quench-hardened and low-temperature tempered 100CrMnSi6-4 bearing steel, was used [31]. Despite the difficulties with surface activation of the austenitic steels, many typical thermo-chemical processes also resulted in the formation of wear resistant surface layers. The sintered WC-Co ball [35], Al_2_O_3_ ball [38] or WC (tungsten carbide) ball [40] became the counter-samples during the wear tests of powder-pack borided (P-PB) layers using “ball-on-disc” methods. The scratch test [37] was employed to assess the wear behavior of boride layers based on the obtained coefficient of friction (CoF). The three-body abrasion tests were also performed after P-PB [39]. In this case, AISI 52100 steel balls rotated against a flat borided sample in the presence of abrasive slurry. The “pin-on-disc” method with AISI 52100 steel pins was used to evaluate the wear resistance of powder-pack borided layer [41]. The hybrid layers fabricated using P-PB followed by powder-pack chromizing (P-PCr) or P-PCr followed by P-PB were also studied [41]. Whereas the wear behavior of some powder-pack borided layers [33,34,36] as well as the layers borided in liquid media [42,43] was not studied. During the tribocorrosion tests of low-temperature gas nitrided layers, the “ball-on-disc” technique was applied using alumina ball as a counter-sample [44]. The similar tests were also carried out after low-pressure and low-temperature gas nitriding (LTGN) of high-velocity oxy-fuel (HVOF) sprayed 316L coating [45] in order to investigate the wear resistance of the layers produced. The nitrided layers, produced by high-temperature gas nitriding (HTGN) [46,47] as well as by nitriding in liquid media [48,49] were not subjected to wear tests. The wear behavior of powder-pack carburized austenitic steel was not provided [50,51]. The wear tests of low-temperature carburized layer, produced on 316L steel in unknown medium [52], were carried out using the “block-on-ring” method with the two various ring-shaped counter-specimens: untreated 316L steel and the same way carburized 316L steel. The “ball-on-disc” technique was employed in order to evaluate the wear behavior of thin TiN coatings, fabricated on the 316L substrate by PVD technique, using WC-Co [53] and silicon nitride (Si_3_N_4_) [54] balls as counter-specimens. Some of the surface layers, produced on austenitic 316L steel by laser surface alloying (LSA), were also subjected to wear tests [57,60,61,62]. The “pin-on-disc” method with WC-Co drill as counter-specimen was used in order to assess the wear behavior of laser-alloyed layers with Cr_3_C_2_ chromium carbides [57,61], TiC titanium carbides [61], a mixture of Cr_3_C_2_ and Cr or mixture of Ti and SiC silicon carbide [57]. The wear resistance of laser-alloyed layer with boron was studied using “block-on-ring” technique in which the counter-sample was composed of S20S (WC-Co) sintered carbide [60]. The “ball-on-disc” method with the use of ZrO_2_ ball as a counter-specimen was applied in the paper [62], in which the alloying material consisted of 80 wt.% Cr and 20 wt.% CrB_2_ powders.

It was difficult to compare the results of wear tests of the surface layers, produced in austenitic steel using the various techniques, because of the differences in the techniques of wear behavior investigation, the methods of its evaluation as well as the load used. Therefore, the most appropriate method of comparing the effects of different surface treatment seemed to be the indication how many times the wear resistance had increased in comparison with untreated austenitic steel using specific evaluation method. The results of such considerations are shown in Table 3 for physical techniques of formation of surface layers on austenitic steel. Volumetric wear simply meant a loss of the specimen volume during the wear test. The specific wear rate was usually measured by the ratio of the sample volume loss to the product of the applied load and sliding distance. The coefficient of friction (CoF) was defined as the ratio of applied load to the frictional force. By mass loss, the mass loss of the specimen during the wear test was understood. The mass wear intensity factor and relative mass loss were defined previously in the present study. 

The laser-alloyed layers, presented in this study, were characterized by 15–26-times lower mass wear intensity factors and 5–26-times lower relative mass loss in comparison with untreated 316L steel. The wear tests were carried out using “block-on-ring” technique in which ring-shaped specimens and block-shaped counter-specimens were used. It seemed that the more comparable results could be obtained in the case of the surface layers examined with the use of the same “block-on-ring” technique, i.e., low-temperature plasma gas-nitrided [2,3], high-temperature plasma gas-nitrided [3] or plasma paste borided [31] layers. However, some differences characterized these wear tests in comparison with the tests, reported by the present work. First of all, the samples were prepared in the shape of “blocks”, and the ring-shaped counter-samples were made of 100CrMnSi6-4 bearing steel of a hardness of 60 HRC [2,3] or 64 HRC [31]. The differences in the load occurred. Plasma paste borided specimens [31] were investigated using lower load (19.6 N) in comparison with the present study (49 N). In this case, the same methods of evaluation of wear resistance were used, i.e., mass wear intensity factor (*I_mw_*) and relative mass loss. PPB process resulted in 4-fold reduction in the *I_mw_* value and 6-fold reduction in relative mass loss compared to the untreated 316L steel [31]. The effect of PPB on the wear was diminished in comparison with laser-alloyed layers. In the case of the specimens, subjected to LTPGN [2,3] or HTPGN [3] processes, only the load per unit of the friction surface (400 MPa) was provided. However, it was difficult to determine what area was taken into account when calculating this value. Additionally, the oil Lux 10 was used as a lubricant, and the effect on the wear was analyzed using the volumetric wear. The increase in wear resistance of these layers was extremely high, obtaining the volumetric wear up to 371 (LTPGN) or even 920-times lower (HTPGN) than that of untreated austenitic 316L steel [2,3]. 

In the case of wear tests of the surface layers, produced by LTPGN, LTPGNC, HTPGN, CPEN and LTPGC processes, the “ball-on-disc” technique was most popular [5,7,9,15,16,23,26,27]. The volumetric wear, measured after LTPGN and HTPGN under the load of 8.3 N, was 40-times and 2.5-times lower, respectively, when compared to 316L steel without surface treatment [5]. The values of specific wear rate and coefficient of friction, measured at the load of 10 N, were used in order to evaluate the wear behavior of the surface layers, produced by LTPGN or LTPGNC processes [7]. The results confirmed 29-fold and 23-fold reduction in specific wear rate of low-temperature plasma gas nitrided and low-temperature plasma gas nitrocarburized layers, respectively. However, the effect of such processes on coefficient of friction was ambiguous and relatively slight in comparison with untreated steel, obtaining values 1.015-times higher or 1.063-times lower after LTPGN and LTPGNC, respectively. The values of volumetric wear and CoF, measured at a load of 20 N in the case of low-temperature plasma gas nitrided layer [9], were 13.5-times and 1.063-times lower, respectively, than the values characteristic of the austenitic steel without surface treatment. The same methods of wear evaluation were used after LTPGN process, reported in the paper [23]. In this case, at the load of 10 N, the volumetric wear was 1.47-times lower, and coefficient of friction was 1.26-times lower compared to the untreated steel. The tribocorrosion tests at a load of 20 N indicated the 10-fold reduction in volumetric wear after LTPGC process [27]. The specific wear rate was determined in order to evaluate the effect of various HTPGN processes on wear using the load of 3 N [15]. Its measured values were from 175- to 650-times lower in comparison with the untreated austenitic steel. Whereas the coefficient of friction, measured after CPEN (cathodic plasma electrolytic nitriding) at load of 3 N, was only 1.6-times lower [16]. The particular method of wear evaluation was used in the case of LTPGC process, reported by the paper [26]. The percentage of volume removed was defined as the ratio of the volume loss per unit of length of carburized sample to the volume loss per unit of length of untreated sample. The percentage of volume removed, thus calculated, was equal to 1 for the material without surface treatment, and 10-times lower for the carburized layer [26]. 

The “ball-on-disc” technique was also used during the wear tests of hybrid layers, produced by hybrid treatment [19,21,22,23] and TiN coatings [53,54]. LTPGN followed by MAIP (multi-arc ion plating) resulted in the greater effect on wear at the same load (10 N) than only LTPGN process, obtaining 11.2-times lower volumetric wear and 2.25-times lower friction coefficient compared to the untreated steel [23]. The wear evaluation of the combined processes consisting in SP (shot peening) followed by LTPGC and LTPGN processes at 475 °C was performed using the volumetric wear [19]. Its value, measured at a load of 15 N, was up to 65-times lower than that characteristic of the austenitic steel without surface treatment. Although the volumetric wear of the surface layers, produced by SP process followed by LTPGN, was not specified in Table 3, it was reduced up to 39-times [19]. The specific wear was measured at a load of 1.96 N during the wear tests of other hybrid processes that consisted of: CS (cold spraying) of 316L powder and LTPGN or LTPGC, CS of such a powder followed by LTPGC and LTPGN or followed by a simultaneous low-temperature plasma gas nitrocarburizing (LTPGNC) process [21]. Among these processes, two of them were characterized by the greatest effect on reducing the wear. In the case of CS of 316L steel followed by LTPGN or CS of 316L steel followed by LTPGC and LTPGN, the values of specific wear rate were 10–26- or 9–26-times lower when compared to the untreated 316L steel, respectively. The same method of wear evaluation was reported in the paper [22] regarding the combined processes consisting in LMD (laser metal deposition) of 316L steel and nickel powders on the surface of AISI 304 austenitic steel prior to LTPGN [22]. The values of specific wear rate were from 56 to 175-times lower in comparison with this steel without surface treatment. TiN coating, produced by PVD technique, was characterized by 2.52-fold reduction in mass loss per friction distance and 1.37-fold reduction in coefficient of friction at a load of 5 N [53]. Similar TiN coating, reported in the paper [54], indicated the specific wear rate up to 13.67-times lower and CoF values 1.11–5-times lower than the values characteristic of the untreated 316L steel. 

The “pin-on-disc” technique was used less often for layers or coatings produced on austenitic steel by physical methods. In the Table 3, only two of the physical techniques of the surface layers formation were taken into account. The wear of the low-temperature gas nitrided layer was examined under the highest load of 100 N [8]. The effect of this surface treatment on the wear was ambiguous. The mas loss was 4.6–91-times lower, and the coefficient of friction was 1.075–1.47-times higher in comparison with the untreated 316L steel [8]. In the case of LSA with the mixture of Cr_3_C_2_ and Cr powders, specific wear rate in deionized water was characterized by 1.29-fold reduction compared to the untreated 316L steel. Whereas the coefficient of friction did not change significantly during the wear tests at a load of 5 N [57]. In the paper [61] that was published by the same authors, the wear behavior of the laser-alloyed layers with Cr_3_C_2_ or TiC was studied at the same load (5 N). The specific wear rate was up to 1.66 and 5.93-times lower, respectively, and CoF values were slightly higher or lower in comparison with untreated 316L steel. The wear behavior after the hybrid process, consisting in SLM followed by LTPGN, was evaluated using the specific wear rate and coefficient of friction [20]. In this case, the “pin-on-disc” technique was also applied under a load of 10 N. However, the results were not compared to the 316L steel without surface treatment. Unfortunately, among the physical techniques analyzed were those for which no wear tests were carried out. Therefore, it was not possibility to compare the wear behavior of the surface layers produced by LTPGN and LTPGN with active screen [10], HTPGN [14], SP + LTPGN [17], LTPGC [28] or LSA with NiCoCrB [59].

The thermochemical techniques of producing surface layers on austenitic steel were compared in a similar way (Table 4). For similar reasons, the amount of times the wear resistance of the layers increased compared to the untreated steel was also analyzed. Simultaneously, the wear test technique, load and methods of wear evaluation were indicated in Table 4. As was the case with the physical techniques for the formation of the surface layers, among the methods of wear evaluation used, the volumetric wear, specific wear rate, coefficient of friction (CoF) and mass loss of the samples were the most commonly used. Additionally, the wear area and wear depth were used in order to evaluate the wear behavior of the surface layers [45]. The wear area corresponded to the worn surface of the surface layer, and wear depth denoted the maximal depth of the wear scar formed on the investigated surface.

The surface layers, fabricated by powder-pack boriding (P-PB), were usually subjected to wear tests using the “ball-on-disc” technique [3,38,39,40] or “pin-on-disc” technique [41]. The specific wear rate and coefficient of friction were measured at a load of 5 N after the P-PB process, as reported in the paper [35], obtaining the values which were 4–9-times lower and 1.31–4.13-times lower, respectively, when compared to the untreated 316L steel. The same methods of wear evaluation were used in the paper [38] at loads of 5 N or 20 N. However, in this case only the specific wear rate was compared to the 316L steel without surface treatment. Its values were 38–62-times lower, depending on the load and sliding distance used. The coefficients of friction were measured only for the borided layer, obtaining values of 0.55 and 0.65 at load of 5 N and 20 N, respectively. Micro-abrasion wear resistance of powder-pack borided 316L steel was studied in the paper [39] using abrasive slurry, consisting of SiC particles (4–5 μm) dissolved in a 20% volume proportion with deionized water. The specific wear rate of borided layer, measured at the load of 0.2 N, was 1.53-times lower than that of the austenitic steel without surface treatment. The wear resistance of powder-pack borided 316L steel [40] was 15–59-times lower, taking into consideration the values of specific wear rate at a load of 5 N. Whereas the CoF values were 2.03–2.68-times lower, depending on the boriding parameters (temperature and time). The volumetric wear was not compared to the untreated 316L steel [40].

The “pin-on-disc” technique at loads of 95 N and 125 N was used in order to evaluate the wear behavior of powder-pack borided layer as well as the hybrid layers, produced by P-PB followed by P-PCr or P-PCr followed by P-PB [41]. In this case, the two methods of wear evaluation were used: mass loss and coefficient of friction. The greatest increase in wear resistance was recorded for the borided and then chromized samples. Their mass loss indicated 11-fold and 3.71-fold reduction in the mass loss at loads of 95 and 125 N, respectively. The only borided layer was characterized by 4.75-times and 3-times diminished mass loss in comparison with the untreated 316L steel at the same loads. The effect of P-PCr followed by P-PB on the wear was smaller. The values of mass loss were 2- or 1.81-times lower, compared to the austenitic steel without surface treatment. The influence of the surface treatment used on the coefficient of friction was ambiguous [41]. In the case of powder-pack borided layer, the values of CoF, measured at loads of 95 N and 125 N, were 1.055-times and 1.15-times higher, respectively, when compared to untreated 316L steel. The P-PCr process followed by P-PB influenced the coefficient of friction this way that its value was 1.22-times higher at a load of 95 N and nearly the same, if it was measured at a load of 125 N. It seemed that the more advantageous effect on CoF values was obtained after the hybrid treatment, consisting in P-PB followed by P-PCr. Although the coefficient of friction was 1.17-times higher at a load of 95 N, its value was 1.08-times lower at a load of 125 N compared to the untreated 316L steel [41].

The “ball-on-disc” technique was used during the tribocorrosion tests of low-temperature gas nitrided layers in 3.5% NaCl solution [44]. The total mass loss of the specimens resulted from the chemical and mechanical wear. If the specimens were subjected to the load of 2 N, the LTGN process caused 5–70-times the lower mass loss in comparison to the untreated 316L steel, depending on the applied potential. At the load of 10 N, its value was even 1.56-times higher when compared to the austenitic steel without surface treatment. This effect was observed because the S-phase layer was worn through at increased load. However, the coefficient of friction, measured at 10 N, was approximately 1.11-times lower than that of untreated steel [44]. The effects of LTGN process on the wear of previously HVOF-sprayed 316L steel were studied using the “ball-on-disc” technique and “reciprocating ball-on-plane” technique [45]. The wear areas and wear depths were measured after “ball-on-disc” test under a load of 20 N. When compared to the untreated austenitic steel, their values were 2.07–9-times lower and 1.74–5.53-times lower, respectively. During the “reciprocating ball-on-plane” tests, the volumetric wear and wear depth were used in order to evaluate the wear behavior of the specimens. The low-temperature gas nitrided specimens were characterized by 1.75–2.92 and 1.42–2.16-times diminished volumetric wear and wear depth, respectively.

The effect of low-temperature carburizing on the wear of austenitic steel was studied using the “block-on-ring” technique at a load in the range from 5 to 25 N [52]. The volumetric wear of the specimens, subjected to LTC process, was up to 2-times lower in comparison with untreated 316L steel. Some of the previously mentioned surface layers, produced using P-PB [33], SMAT (surface mechanical attrition treatment) followed by P-PB [34], boriding in liquid medium [42] or nitriding in liquid medium [49], were not subjected to wear tests. The results of very-interesting scratch tests, carried out after P-PB or P-PB followed by diffusion annealing [37], were difficult to compare to the other techniques of wear behavior evaluation. 

Summarizing, the laser-alloyed layers, studied in the present work at a relatively high load of 49 N, were characterized by the relatively significant increase in wear resistance, measured by mass wear intensity factor (*I_mw_*) and relative mass loss (Δ*m*/*m_i_*), when compared to the majority of the other physical and thermochemical techniques of the surface treatment. The use of a relatively high load was possible due to the high depths of the laser-alloyed layers produced. In the case of other techniques of surface treatment, the greater effect on wear reduction was usually obtained at much lower load [5,7,15,19,22,38,40] or at the load which was difficult to determine [2,3]. The higher load was reported only during the wear tests of low-temperature plasma gas nitrided layer [8] and the surface layers which were fabricated by P-PB, P-PB followed by P-PCr or P-PCr followed by P-PB [41]. Only the results of the paper [8] indicated the comparable or even greater effect of the surface layer on the wear behavior of austenitic steel.

### 3.3. Wear Mechanisms

The wear mechanism of laser-alloyed layers with boron as well as with boron and selected metallic elements was studied based on the SEM observations of the worn surfaces of the specimens and counter-specimens (S20S sintered carbides) and EDS patterns of selected elements on these surfaces. The same techniques were previously used in order to identify the wear mechanism of laser-borided layer and untreated 316L steel [60]. The worn surface of the laser-borided layer with a dilution ratio of 0.37 indicated, firstly, the intensive abrasive wear represented by the characteristic shallow grooves. Simultaneously, the EDS patterns of selected elements on the worn surfaces indicated the obvious signs of oxidative wear of laser-borided specimen as well as the effects of adhesive wear on the worn surface of the counter-specimen [60]. On the other hand, the worn surface of austenitic steel 316L without surface treatment was characterized by obvious signs of strong plastic deformation as well as abrasive and adhesive wear, as indicated by deep grooves and adhesive craters. The effects of adhesion were confirmed by the EDS patterns of selected elements on the worn surface of the counter-specimen after the wear test of untreated 316L steel [60]. 

**Figure 8:**
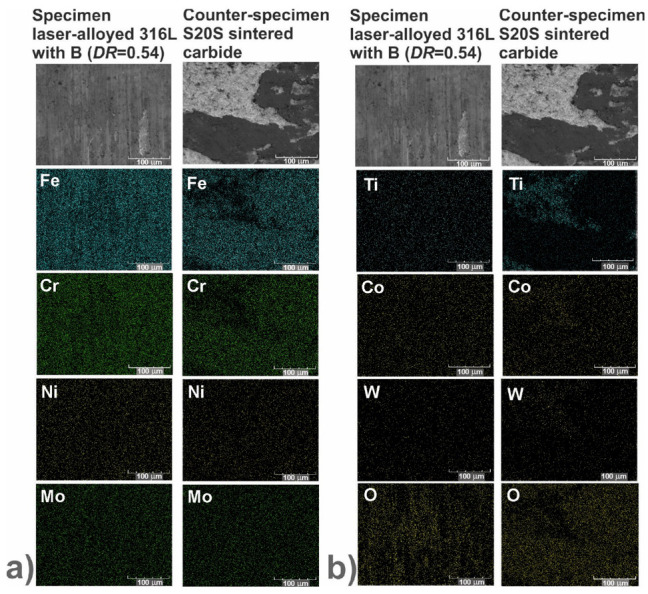
Worn surfaces of laser-borided specimen with a dilution ratio of 0.54 and counter-specimen (S20S sintered carbide). (**a**) EDS patterns of the elements characteristic of the specimen as well as (**b**) the counter-specimen, taking into account oxygen concentration. Two-hour wear test with the change of counter-specimen at every 0.5 h.

The worn surfaces of the laser-borided specimen with a dilution ratio of 0.54 and counter-specimen, made of S20S sintered carbide, are presented in Figure 8. Simultaneously, the EDS patterns of selected elements characteristic of a specimen (Figure 8a) and counter-specimen (Figure 8b) are shown. Additionally, the EDS patterns of oxygen were taken into account in Figure 8b. The worn surface of the laser-borided layer with a higher dilution ratio (0.54) revealed intensive abrasive wear, as indicated by the characteristic shallow grooves (Figure 8). In this case, the reduced concentrations of iron and chromium (Figure 8a) were observed in some darker areas of the sample, which corresponded to a considerably increased oxygen content (Figure 8b). This may indicate the oxidative wear. The light areas with predominant abrasive wear and dark areas with reduced concentrations of titanium, cobalt and tungsten (Figure 8b) as well as increased iron, chromium and oxygen concentrations (Figure 8a) were visible on the surface of mating counter-specimen. This indicated the possibility of adhesive and oxidative wear. The areas of the worn surfaces of the counter-samples with relatively high iron and chromium concentrations overlapped.

**Figure 9:**
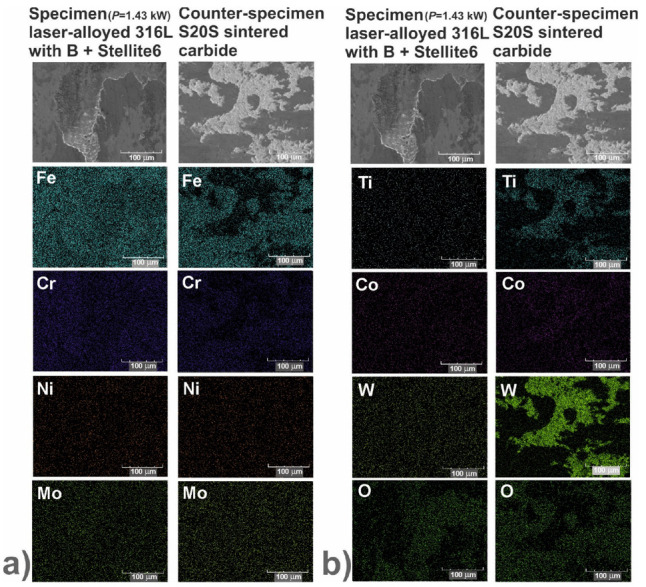
Worn surfaces of laser-alloyed specimen with boron and Sellite-6 using a dilution ratio of 0.41 (*P* = 1.43 kW) and counter-specimen (S20S sintered carbide). (**a**) EDS patterns of the elements characteristic of the specimen as well as (**b**) the counter-specimen, taking into account oxygen concentration. Two-hour wear test with the change of counter-specimen every at 0.5 h.

The worn surfaces of the laser-alloyed specimen with boron and Stellite-6 at a dilution ratio of 0.41 and counter-specimen, made of S20S sintered carbide, were also observed using SEM (Figure 9). The EDS patterns of selected elements characteristic of a specimen and counter-specimen are shown in Figure 9a,b, respectively. Shallow grooves, indicating abrasive wear, as well as small adhesive craters were observed on the worn surface of the laser-alloyed sample. The increased oxygen content in some darker areas of the sample and counter-sample (Figure 9b) demonstrated possible oxidative wear. The increased iron concentration in some areas of the counter-specimen was due to the probable adhesive wear. Usually, it was accompanied by an increased oxygen content, which in turn would confirm oxidative wear. Significantly lower concentrations of tungsten, cobalt and titanium were also observed in the areas where oxides appeared on the worn surface of the counter-specimen. A relatively low concentration of chromium was observed on the worn surface of the specimen. Chromium was also not detected in more quantities on the worn surface of the counter-specimen. This may have indicated its reduced contents close to the surface of the laser-alloyed layer produced.

**Figure 10:**
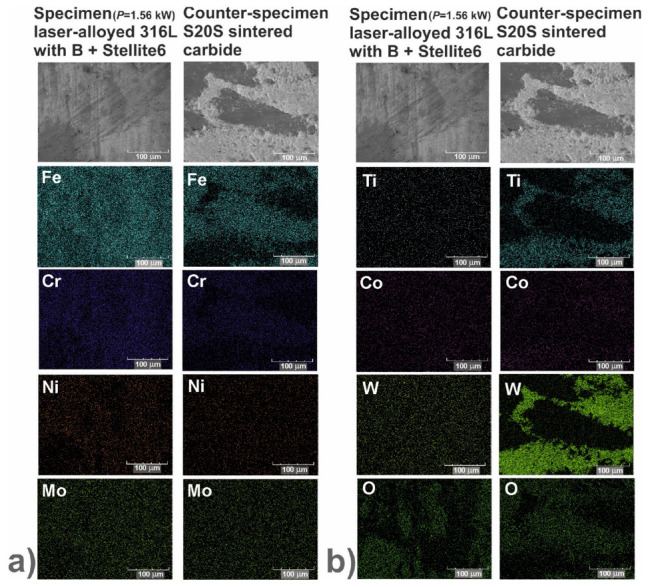
Worn surfaces of laser-alloyed specimen with boron and Sellite-6 using a dilution ratio of 0.48 (*P* = 1.56 kW) and counter-specimen (S20S sintered carbide). (**a**) EDS patterns of the elements characteristic of the specimen as well as (**b**) the counter-specimen, taking into account oxygen concentration. Two-hour wear test with the change of counter-specimen at every 0.5 h.

SEM observations and X-ray microanalysis on the worn surfaces of 316L steel, laser-alloyed with boron and Stellite-6 alloy at higher laser beam power (1.56 kW), and mating counter-specimen (S20S), led to similar conclusions as those formulated with the use of a lower laser beam power (1.43 kW). The worn surfaces of the sample and counter-sample, together with the EDS patterns of distribution of selected elements, are shown in Figure 10. Shallow grooves on the worn surface of the laser-alloyed sample indicated its obvious abrasive wear. Small adhesive craters were also visible. It was likely that oxidative wear also occurred, as evidenced by the increased oxygen content in some darker areas of the specimen and counter-specimen (Figure 10b), as well as by the increased iron concentration on the worn surface of the counter-specimen in these areas (Figure 10a) that could also result from the adhesive wear. As in the previous case, relatively low concentrations of tungsten, titanium and cobalt were observed on the worn surfaces of the counter-samples in the areas in which the oxides appeared. Simultaneously, relatively low chromium concentrations were detected on the worn surfaces of the sample and counter-sample. It was characteristic of both laser-alloyed specimens with boron and Stellite-6 that the increase in cobalt content was not visible on their worn surfaces, despite the presence of cobalt in the alloying material.

**Figure 11:**
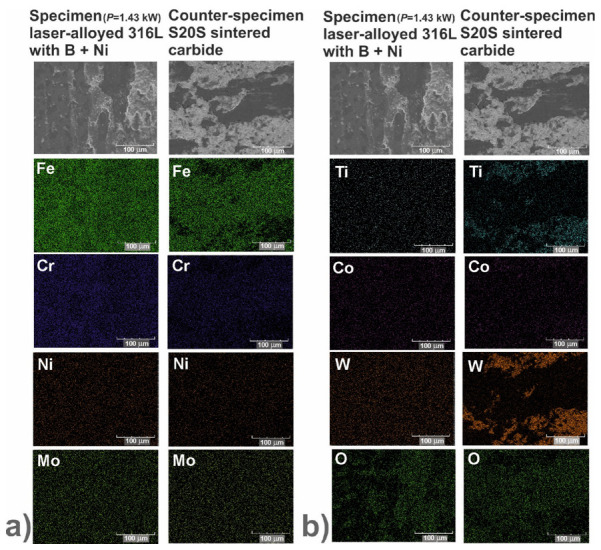
Worn surfaces of laser-alloyed specimen with boron and nickel using a dilution ratio of 0.42 (*P* = 1.43 kW) and counter-specimen (S20S sintered carbide). (**a**) EDS patterns of the elements characteristic of the specimen as well as (**b**) the counter-specimen, taking into account oxygen concentration. Two-hour wear test with the change of counter-specimen at every 0.5 h.

Figure 11 shows the worn surfaces of the laser-alloyed specimen with boron and nickel at a dilution ratio of 0.41 and the counter-specimen with distribution of the analyzed elements by the EDS method. Similar wear mechanisms have been observed as in previous cases. Shallow grooves on the worn surface of the laser-alloyed sample showed abrasive wear and small craters indicated adhesive wear. The increased oxygen content was observed in darker areas of the sample and counter-sample (Figure 11b). Together with the increased iron concentrations on the worn surface of the counter-specimen, it indicated likely wear by oxidation. Such an increased iron content on the counter-sample may also have been partly due to adhesive wear. Simultaneously, the areas with oxides on the worn surface of the sample indicated a diminished iron content. In the probable areas of occurring oxides, significantly reduced concentrations of tungsten, titanium and cobalt were also visible on the worn surface of the counter-sample. There was a relatively low concentration of chromium on the worn surface of the specimen as well as counter-specimen. As in previous cases, this could indicate its reduced content in the surface zone of the laser-alloyed layer produced.

**Figure 12:**
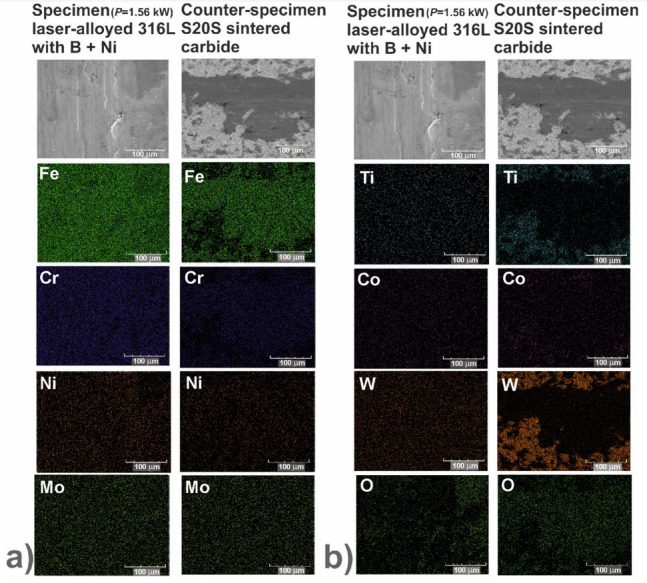
Worn surfaces of laser-alloyed specimen with boron and nickel using a dilution ratio of 0.48 (*P* = 1.56 kW) and counter-specimen (S20S sintered carbide). (**a**) EDS patterns of the elements characteristic of the specimen as well as (**b**) the counter-specimen, taking into account oxygen concentration. Two-hour wear test with the change of counter-specimen at every 0.5 h.

The worn surfaces of 316L steel, laser-alloyed steel with boron and nickel at a dilution ratio of 0.48, and mating with it counter-sample are shown in Figure 12 together with EDS patterns of distribution of selected elements. The wear mechanisms of this laser-alloyed specimen were similar to the previous surface layers analyzed. Shallow grooves on the worn surface of the laser-alloyed sample indicated abrasive wear, while the presence of small craters confirmed adhesive wear. The darkest areas on the worn surfaces of the sample and counter-sample were characterized by an increased oxygen content (Figure 12b), and an increased iron concentration was visible on the worn surface of the counter-sample in these areas (Figure 12a). This indicated the probable oxidative wear. The relatively high iron content on the worn surface of the counter-specimen may also have been partly due to adhesive wear. Simultaneously, the diminished iron contents were detected in the areas with oxides on the worn surface of the specimen. As in the previous cases, the areas of occurring oxides were characterized by diminished tungsten, titanium and cobalt contents on the worn surface of the counter-sample (Figure 12b), and relatively low chromium content was observed on the worn surfaces of the specimen and counter-specimen.

**Figure 13:**
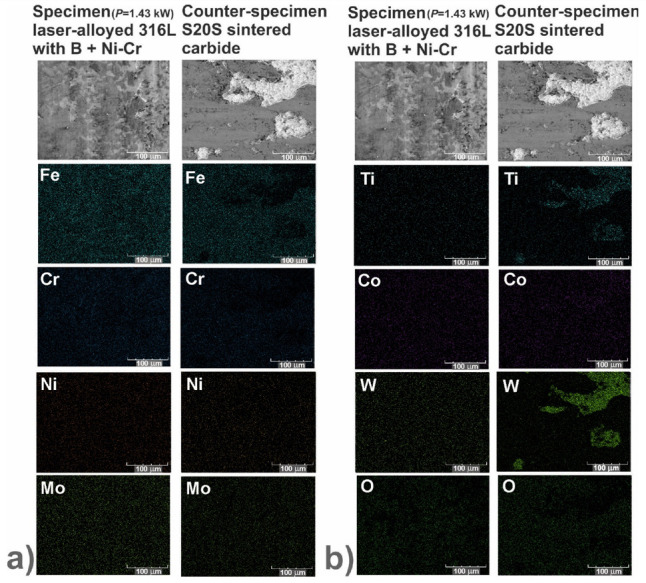
Worn surfaces of laser-alloyed specimen with boron, nickel and chromium using a dilution ratio of 0.43 (*P* = 1.43 kW) and counter-specimen (S20S sintered carbide). (**a**) EDS patterns of the elements characteristic of the specimen as well as (**b**) the counter-specimen, taking into account oxygen concentration. Two-hour wear test with the change of counter-specimen at every 0.5 h.

The worn surfaces of laser-alloyed specimen with boron, nickel and chromium at a dilution ratio of 0.43 and corresponding counter-specimen (S20S sintered carbide), together with EDS patterns of distribution of selected elements, are shown in Figure 13. Similar wear mechanisms were found to be present, as those-identified for laser-alloyed only with boron, boron and Stellite-6 or with boron and nickel. Shallow grooves on the worn surface of the sample were a sign of abrasive wear, and small craters confirmed adhesive wear. The darker areas, observed on the worn surfaces of the sample and counter-sample (Figure 13b) were characterized by an increased oxygen content. The increased iron concentration was also recorded on the worn surface of the counter-sample in these areas (Figure 13a). This indicated a high probability of wear by oxidation. The relatively high iron content on the worn surface of the counter-specimen may also have confirmed adhesive wear. On the other hand, in the areas where oxides appeared, the relatively low iron content was detected on the worn surface of the specimen. In the areas where oxides probably occurred on the worn surface of the counter-sample, tungsten and titanium concentrations were significantly lower. In contrast to the case of laser alloying with the previous types of alloying materials, the worn surface of the sample clearly showed the relatively high chromium concentration, which was also observed on the surface of the counter-sample, probably as a result of the combined oxidative wear and adhesive wear. It is estimated that the reason for this was due to the inclusion of chromium as a part of the alloying material.

**Figure 14:**
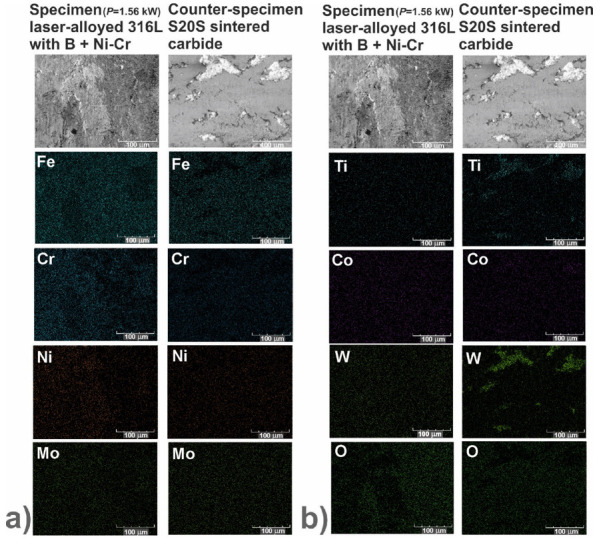
Worn surfaces of laser-alloyed specimen with boron, nickel and chromium using a dilution ratio of 0.49 (*P* = 1.56 kW) and counter-specimen (S20S sintered carbide). (**a**) EDS patterns of the elements characteristic of the specimen as well as (**b**) the counter-specimen, taking into account oxygen concentration. Two-hour wear test with the change of counter-specimen at every 0.5 h.

The similar analysis enabled to identify the wear mechanisms of laser-alloyed specimen with boron, nickel and chromium at a dilution ratio of 0.49. The worn surfaces of the specimen and counter-specimen after wear test as well as their EDS patterns are shown in Figure 14. In general, the same wear mechanisms have been identified as in previous cases, i.e., abrasive wear, adhesive wear and oxidative wear, based on the SEM images of the worn surfaces and EDS patterns obtained.

Summarizing, the wear behavior of all the laser-alloyed alloyed layers, presented in this work, was characterized by a predominant abrasive wear as well as oxidative wear and to a lesser extent observed adhesive wear. The wear mechanisms of other surface layers, fabricated by physical techniques, were identified using various methods and devices, e.g., OM images [5,7,9,27], SEM images [15,23,53,54,60], optical profilometer [7], WDS (wavelength dispersive spectrometer) [15], EDS (energy dispersive spectrometer) [23,54,60] or confocal microscope [16]. Many of these papers analyzed the wear mechanisms under condition of dry friction (unlubricated sliding contact) [5,7,8,9,15,16,19,20,21,22,23,26,31,53,54,60]. Some of them confirmed abrasive, adhesive as well as the oxidative wear with severe plastic deformations in the case of the untreated 316L steel [7,9,15,16,23,27,53,54,60], irrespective of the technique of wear tests and the load used. However, in many papers the wear mechanisms of the surface layers were not studied, assuming their predominant abrasive wear [2,3,8,21,22,26,31,57]. In the case of hybrid surface treatment, consisting in SP (shot peening) followed by LTPGC and LTPGN processes at 475 °C [19], the authors indicated that the thin layer demonstrated possible adhesive wear due to the plastic deformation in the underlying substrate. The thick layer was necessary to avoid this plastic deformation [19].

Some of the surface layers, produced by the physical techniques, were subjected to the detailed analysis of wear mechanisms. They are specified in Table 5. The wear mechanisms of low-temperature or high-temperature plasma gas nitrided layers [5] were studied using optical microscope (OM). OM images of the worn surfaces revealed the craters with scratching and rolling which confirmed grooving abrasion and rolling abrasion, respectively. The same method of wear mechanism identification was used after wear tests of the specimens subjected to LTPGN and LTPGNC processes [7]. The wear tracks were characterized by shallow grooves and adhesion craters, revealing the abrasive and adhesive wear, respectively. Additionally, the transverse cracks were observed in the wear tracks using optical profilometer what confirmed the plastic deformation of the material underneath the surface layer [7]. OM images of the wear scars on the worn surface of low-temperature plasma gas nitrided layer [9] indicated the shallow grooves, corresponding to abrasive wear. SEM images of wear tracks revealed the shallow grooves, confirming the abrasive wear of high-temperature plasma gas nitrided layers [15]. Simultaneously, the WDS pattern of oxygen demonstrated the increased oxygen concentration, especially in the case of the adjacent areas to the wear tracks. It could confirm the presence of oxides and thus oxidative wear [15].

The confocal microscope was used in order to identify the wear mechanism of the surface layer produced by CPEN [16]. The shallow grooves were observed on its worn surface, revealing the abrasive wear. The wear mechanisms of the layers, fabricated by LTPGN process and LTPGN followed by MAIP (multi-arc ion plating), were identified using SEM images and EDS X-ray microanalysis [23]. SEM images revealed slight scratches and craters (dark pitches) on the worn surfaces of these layers, corresponding to abrasive and adhesive wear, respectively. The increased oxygen content, measured by EDS method, confirmed the presence of oxides on the worn surfaces and thus oxidative wear [23]. The low-temperature plasma gas carburized 316L steel was subjected to tribocorrosion tests in 1 M H_2_S0_4_ and 0.5 M NaCl solutions [27]. Shallow grooves in OM images of worn surfaces confirmed the abrasive wear. Additionally, the signs of general corrosion (oxygen evolution) in 1 M H_2_S0_4_ and crevice corrosion in 0.5 M NaCl indicated corrosive wear. No pitting corrosion was revealed on the worn surfaces. TiN coatings, produced by PVD technique, were characterized by abrasive wear [53] based on the SEM images of worn surface. In the case of the similar coatings, reported in the paper [54], the SEM images of the worn surfaces revealed abrasive and adhesive wear [54]. Simultaneously, based on the EDS X-ray microanalysis, the increased oxygen concentration was detected on the worn surfaces. It confirmed the presence of oxides and thus the oxidative wear [54]. The wear mechanisms of laser-borided layer, reported in the previous study [60], were the same as those-identified for the laser-alloyed layers in this work.

The wear mechanisms of the surface layers, fabricated by thermochemical treatment, are listed in Table 6. Most of these layers were subjected to wear tests under conditions of dry friction (unlubricated sliding contact) [35,38,40,41,45,52], excluding the powder-pack borided layer [39] and low-temperature gas nitrided layer [44]. The similar methods and devices were used in order to identification of wear mechanism, e.g., SEM images of the worn surfaces [35,40,44,45], EDS analysis [35], optical profilometer [38,39] or analysis of percentage of SiC particles in the abrasive slurry [39]. SEM images of the worn surface of powder-pack borided layers revealed deep scars and adhesion craters after wear tests under conditions of dry friction as well as in simulated body fluid (SBF) [35]. They confirmed the abrasive and adhesive wear mechanism of such a layer, respectively. The worn surfaces were substantially oxidized. The EDS analysis revealed the increased oxygen content on the worn surfaces what confirmed oxidative wear. This wear mechanism predominated, if the wear test was performed in SBF (simulated body fluid) medium [35]. The grooves and material shedding (debris) on the worn surface of powder-pack borided layers confirmed their abrasive wear [38]. Simultaneously, based on the paper [64] and intergranular or surface cracks, the authors suggested pitting and cracking as well as oxidative wear. The surface layer, produced by P-PB, was subjected to the wear test in the abrasive slurry, consisting of SiC particles (4–5 μm) dissolved in a deionized water [39]. The high percentage of abrasive SiC particles in the slurry and relatively low load favored rolling abrasion. Simultaneously, the low percentage of abrasive SiC particles in the slurry and relatively high load caused grooving abrasion. SEM images of the worn surface of powder-pack borided layer revealed micro-cutting and micro-plowing [40]. It confirmed the abrasive wear. On the other hand, micro-cracking and micro-fatigue revealed the suffer fracture and pitting, respectively. The wear scars were also characterized by plastic deformations as well as debris-adhesion and delaminations which could indicate the adhesive wear [40]. The tribocorrosion test in 3.5% NaCl solution was carried out after LTGN process [44]. In the SEM images of worn surfaces of low-temperature gas nitrided layer the abrasion marks and micro-pits were clearly visible. They confirmed the abrasive and corrosive wear, respectively. The wear mechanisms of low-temperature gas nitrided layer, produced in HVOF-sprayed 316L steel, were examined using SEM images of the worn surface [45]. The deep grooves and breakout of hardened spray particles revealed abrasive wear of such a layer. 

In some papers, reporting the wear behavior of the surface layers produced by thermochemical treatment, the wear mechanisms were not studied in more details [41,52]. In the case of P-PB processes and hybrid treatment, consisting in P-PB followed by P-PCr or P-PCr followed by P-PB, the wear mechanisms were not identified [41]. In general, the abrasive wear was assumed. It was observed that the mass loss of the counterpart pins increased with the applied load. This happened due to more sever engagements at the contact surfaces, and subsequently heat accumulated locally at the contact surfaces. This situation increased the possibility of adhesive wear [41]. The wear mechanism of low-temperature carburized layer was also not identified [52]. It was concluded that only the carburized layer resulted in the improved resistance of the surface to plastic deformation and abrasion, as well as to limit adhesion between the mating surfaces.

### 3.4. Corrosion Resistance

Corrosion resistance tests were conducted using ATLAS 0531 electrochemical unit and impedance analyzer in 3.5% NaCl and 0.5 M H_2_SO_4_ solutions on the flat samples (Figure 1) which were laser-alloyed with boron and selected metallic elements (Stellite-6, Ni, Ni-Cr) at laser beam power of 1.43 kW and laser-alloyed with boron only at laser beam power of 1.82 kW. The test results of the laser-alloyed layers were compared with those-obtained from untreated 316L steel. The corrosion tests were conducted at 22 °C (295 K) in two corrosive media: 3.5% aqueous NaCl solution and 1 M H_2_SO_4_ solution. The potential ranged from −2 to 2 V, with a potential change rate of 0.5 mV/s.

**Figure 15:**
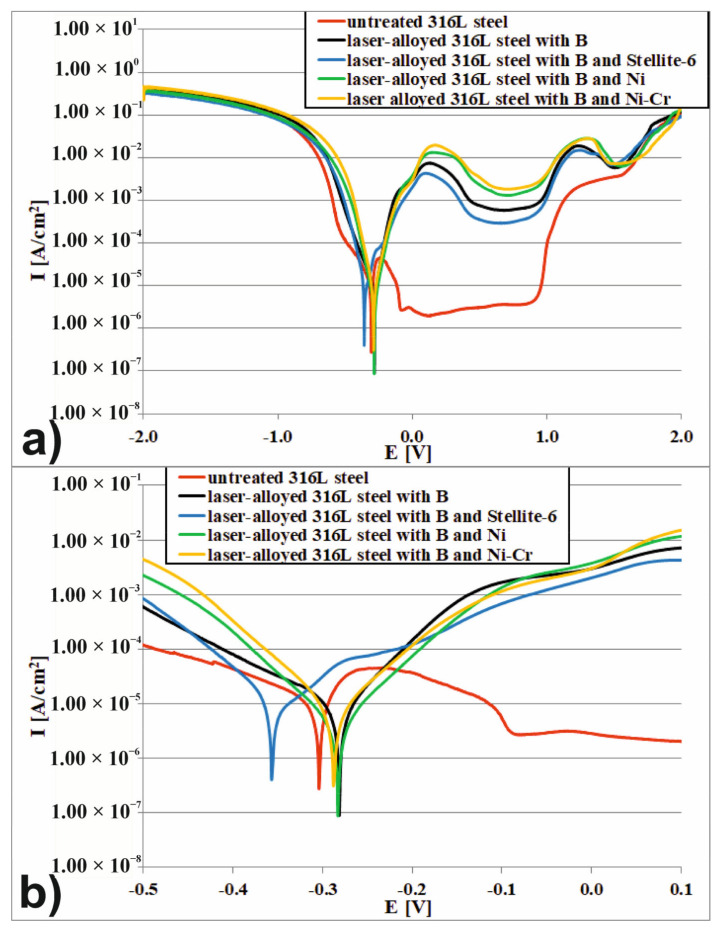
Polarization curves obtained in 1 M H_2_SO_4_ corrosion medium by the potentiodynamic method for laser-alloyed layers with boron and selected metallic elements produced on 316L steel using a laser beam power of 1.43 kW compared to laser-alloyed steel with only boron with a dilution ratio of 0.54 and 316L steel without treatment. Potential range from −2 to 2 V, potential change rate 0.5 mV/s. The entire polarization curves (**a**) and magnification of the area in which corrosion potential and corrosion current density were determined (**b**).

The polarization curves in 1 M H_2_SO_4_ solution are shown in Figure 15, and the calculated values of corrosion potential *E_corr_* and corrosion current density *I_corr_* are specified in Table 7. It was expected that the single-phase austenitic structure of untreated 316L steel will be characterized by the greatest corrosion resistance, while the composite boride layers produced by laser alloying would have a slightly lower corrosion resistance due to their multiphase microstructure. The laser-alloyed layer with boron and Stellite-6 was undoubtedly characterized by a worst corrosion resistance, because the corrosion potential for this sample was the most negative (*E_corr_* = −356.88 mV) and at the same time the corrosion current density was the highest (*I_corr_* = 15.6 × 10^−6^ A/cm^2^), indicating that the largest amount of ions of this material was being digested into the electrolyte. It turned out that the other laser-alloyed layers showed a very-similar corrosion potential (between −286.89 mV and −279.59 mV), which with also a slight difference in the corrosion current density (from 4.5 × 10^−6^ to 9 × 10^−6^ A/cm^2^) showed that their corrosion resistance was comparable. The untreated 316L steel was characterized by a slightly more negative corrosive potential (−304.11 mV) and a slightly higher corrosion current density (10.1 × 10^−6^ A/cm^2^) compared to laser-alloyed layers with boron, boron and nickel or boron, nickel and chromium.

However, significant differences in the course of polarization curves were visible. For the 316L steel without surface treatment, the region of active digestion was much smaller compared to the laser-alloyed samples, indicating better corrosion resistance. Simultaneously, the region of primary passivation for untreated 316L steel was approximately twice as wide (within the range of potentials) compared to all the laser-alloyed samples. It was also important that for untreated material, the passive region was accompanied by a very low current density (2.5 × 10^−6^ A/cm^2^) compared to all the laser-alloyed layers, which were characterized by the current density in passive region from approximately 3 × 10^−4^ A/cm^2^ for the laser-alloyed layer with boron and Stellite-6 up to about 2 × 10^−3^ A/cm^2^ for the laser-alloyed layer with boron, nickel and chromium. This confirmed the very good corrosion resistance of 316L steel in 1 M sulfuric acid environment. The relatively narrow passive region, observed in the case of laser-alloyed specimens, could be related to the resistance of borides to oxidation. Analysis of the surfaces of the specimens after corrosion tests revealed clear pits in all the laser-alloyed layers, while the surface of untreated 316L steel indicated more uniform corrosion. In conclusion, as expected, the untreated 316L austenitic steel was characterized by the best corrosion resistance in 1 M H_2_SO_4_. The slightly less corrosion resistance was revealed for laser-alloyed layers with boron, boron and nickel as well as boron, nickel and chromium. By far the worst corrosion resistance was demonstrated by the laser-alloyed layer with boron and Stellite-6.

**Figure 16:**
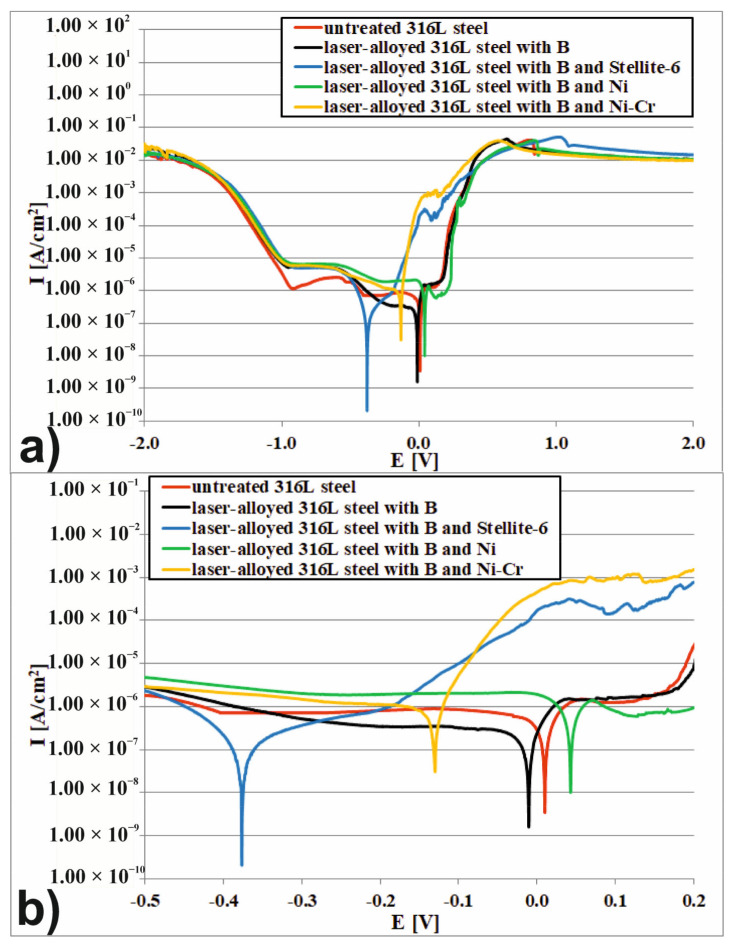
Polarization curves obtained in 3.5% NaCl corrosion medium by the potentiodynamic method for laser-alloyed layers with boron and selected metallic elements produced on 316L steel using a laser beam power of 1.43 kW compared to laser-alloyed steel with only boron with a dilution ratio of 0.54 and 316L steel without treatment. Potential range from −2 to 2 V, potential change rate 0.5 mV/s. The entire polarization curves (**a**) and magnification of the area in which corrosion potential and corrosion current density were determined (**b**).

The results of the studies in 3.5% NaCl water solution are shown in Figure 16 and Table 8. Undoubtedly, the laser-alloyed layer with boron and Stellite-6 was characterized by the least corrosive resistance. Its corrosion potential was the most negative (*E_corr_* = −371.19 mV) and the region f active digestion was the widest, although at the same time the corrosion current density was quite low (*I_corr_* = 64.6 × 10^−8^ A/cm^2^). The sample after laser-alloying with boron, nickel and chromium had the second negative value of corrosive potential (−132.84 mV) and the highest value of corrosion current density (22.5 × 10^−7^ A/cm^2^), indicating digestion of the relatively large amount of material ions into electrolyte with a much narrower active region. By far the highest corrosion resistance in 3.5% NaCl solution was demonstrated by the laser-alloyed specimens exclusively with boron, boron and nickel as well as untreated 316L steel. Laser-alloyed 316L steel with boron and nickel showed the most positive corrosive potential (*E_corr_* = 43.32 mV) and simultaneously the second in order corrosion current density (*I_corr_* = 99.7 × 10^−8^ A/cm^2^). The untreated 316L steel also had positive corrosion potential (*E_corr_* = 9.87 mV) and lowest corrosion current density (*I_corr_* = 52.2 × 10^−8^ A/cm^2^). Laser-alloyed 316L steel only with boron was characterized by a negative corrosion potential (*E_corr_* = −10.97 mV), but a very-low corrosion current density (*I_corr_* = 63.8 × 10^−8^ A/cm^2^). In the latter three cases, the comparable regions of active digestion and similar current densities were found in the region of primary passivation. The clear pits on the surfaces of all the laser-alloyed layers were visible after corrosion tests, especially, in the case of laser-alloyed specimen with B and Stellite-6 which revealed brown color of these pits. Summarizing, the best corrosion resistance in 3.5% NaCl solution was obtained in the case of austenitic steel 316L without surface treatment and laser-alloyed layers exclusively with boron or boron and nickel. By far the worst corrosion resistance was demonstrated by the laser-alloyed layer with boron and Stellite-6.

Based on the potentiodynamic tests of corrosion resistance in both media (1 M H_2_SO_4_ and 3.5% NaCl), the best corrosion behavior was obtained for the materials as follows: untreated austenitic 316L steel (according to expectations) as well as laser-alloyed layers with boron and nickel or exclusively with boron. Taking into account the tests of microstructure, microhardness, wear resistance and corrosion resistance, the laser-alloyed layer with boron and nickel should be considered as the best layer produced in the 316L steel by laser surface alloying.

The determined values of corrosion potential *E_corr_* and corrosion current density *I_corr_* of the untreated [2,4,5,6,7,8,10,14,15,16,17,19,24,27,35,36,41,44,52], as-sprayed [21] and as-deposited [22] austenitic 316L steels are specified in Table 9 based on the other papers. In many of them, the corrosion current density was not calculated [4,5,6,8,10,14,15,17,19,21,27,52]. Simultaneously, some of these papers did not report the corrosion potential [4,6,10,14,17,21,27,44] directly. In these cases, the values of *E_corr_* were estimated based on the polarization curves and were marked with asterisk in Table 9. The differences in the media used as well as the various parameters of the tests influenced the obtained results. 

Some of these media corresponded to those-applied in the present study, reporting the corrosion behavior of 316L steel in 3.5% NaCl [9,14,15,16,21,22,41,44,52] and 1 M H_2_SO_4_ [27] solutions. The corrosion current density (*I_corr_*), calculated in some papers [9,16,41] after tests in 3.5% NaCl, was nearly the same (55 × 10^−8^, 54.7 × 10^−8^ and 52.68 × 10^−8^ A/cm^2^, respectively). However, in contrary to the present work, the corrosion potential (*E_corr_*) was slightly negative, obtaining the values of −241 mV, −290 mV and −163.1 mV, respectively. The similar value of *E_corr_* (−275 mV) was estimated based on the polarization curve shown in the paper [14]. In the papers [44,52], the less negative values of corrosion potentials were reported (−160 mV and −150 mV, respectively). Simultaneously, the results of the paper [44] indicated the relative high corrosion current density (1.42 × 10^−3^ A/cm^2^). Sometimes, the corrosion potential of untreated 316L steel in 3.5% NaCl solution obtained more negative value (−946 mV) [15]. The as-sprayed (by CS) [21] and as-deposited (by LMD) [22] 316L steel was also characterized by slightly negative values of *E_corr_*, i.e., −270 and −249 mV, respectively. The corrosion current density of as-deposited 316L steel [22] was relatively high (1 × 10^−3^ A/cm^2^). The tribocorrosion behavior of untreated 316L steel was studied in 1 M H_2_SO_4_ solution without or with sliding [27]. The corrosion potential was equal to approximately −210 mV without sliding and −280 mV with sliding, being comparable to that-obtained in the present study (−304.11 mV). Summarizing, the values of corrosion potentials and corrosion current densities, obtained in the present work for the untreated 316L steel (Table 7 and Table 8), well corresponded to the other literature data and seemed to be reliable.

It was more difficult to compare the results of the present work to the corrosion behavior of 316L steel in other media, e.g., Ringer’s solution [2,6], aerated 3% NaCl [4,6,8], aerated 0.6 M NaCl [5], 0.5 M NaCl [7,10,27], 5% NaCl [17], natural seawater [24], simulated body fluid [35] and 0.5 M H_2_SO_4_ [19,52]. Usually, the determined or estimated values of corrosion potentials (*E_corr_*) were slightly negative (from −440 mV [27] to −140 mV [7]), except of the untreated 316L steel which was examined in Ringer’s solution [6], aerated 3% NaCl solution [6] and 5% NaCl solution [17] (15, 50 and 410 mV, respectively). The corrosion current density (*I_corr_*) was calculated in the case of the use of Ringer’s solution [2], aerated 3% NaCl [6], 0.5 M NaCl [7] and natural seawater [24]. Its values were equal to 1.5 × 10^−8^, 6.3 × 10^−5^, 8 × 10^−9^ and 4.94 × 10^−6^ A/cm^2^, respectively. The potentiodynamic tests in simulated body fluid (SBF) [35] resulted in *E_corr_* values from −266 to −255 mV. The corrosion current density ranged from 5.8 × 10^−8^ to 7.4 × 10^−8^ A/cm^2^. 

In the other media, the corrosion current density was not calculated. Sometimes, its value was characterized by a general description. In the case of the untreated 316L steel, investigated in aerated 0.6 M NaCl, the *I_corr_* value was comparable to that after LTPGN [5]. In Ringer’s solution, the corrosion current density was slightly higher than that after LTPGN [6], whereas in 0.5 M NaCl the value of *I_corr_* was nearly the same as after LTPGN process [10]. In 5% NaCl, the corrosion current density significantly exceeded 1 × 10^−5^ A/cm^2^, taking into account the whole polarization curve [17]. In the case of 316L steel, subjected to potentiodynamic test in 0.5 M H_2_SO_4_, only the corrosion potential of −373 mV and the passive current density of 6 × 10^−5^ A/cm^2^ were provided [19]. The paper [52] reported the similar values of *E_corr_* and passive current density in the same medium (−360 mV and 5 × 10^−5^ A/cm^2^, respectively). The potentiodynamic tests were sporadically carried out in the other media, such as the 1 M HCl [36], 0.9% NaCl [36], 1 M NaOH [36], 2.5 vol% HCl [52], 3 vol% CH3COOH [52] or 2.5 vol% Oxonia solutions [52]. Their results are also shown in Table 9. The untreated 316L steel was also subjected to immersion test in 5.85% NaCl solution for 60 days [26]. The corrosion behavior was evaluated based on the OM and SEM images of the tested surface. There was no possibility to compare the obtained results to the potentiodynamic tests.

It was difficult to compare the results of the present work to the potentiodynamic tests which were carried out in the case of surface layers produced by other physical techniques (Table 10). The main reason for such a situation was the differences in the media used as well as the various parameters of the potentiodynamic tests. Sometimes, the corrosion current density was not calculated [4,5,6,8,10,14,15,17,19,21,27]. Simultaneously, the corrosion potential was not often reported directly [6,10,14,17,21,27]. Hence, the estimated values of *E_corr_* were given based on the polarization curves. They were marked with asterisk in Table 10. The selection of the medium used usually took into account the predicted application of the considered surface layer. The use of Ringer’s solution indicated the predicted use of the proposed surface layer in biomedical applications [2,6], whereas the natural seawater and NaCl or H_2_SO_4_ solutions [4,5,6,7,8,9,10,14,15,16,17,19,21,22,24,26,27] have been used in probable applications for structural parts in the construction of machinery. The relatively easy comparison was possible only to the layers, produced by LTPGN [9], HTPGN [14,15], CPEN [16] and LTPGC [27] processes as well as hybrid processes, consisting in cold spraying (CS) of 316L steel followed by LTPGN, LTPGC, LTPGC and LTPGN or LTPGNC [21]. The mentioned above layers were subjected to the potendiodynamic tests in 3.5% NaCl [9,14,15,16,21] or 1 M H_2_SO_4_ [27] solutions, i.e., the solutions also used in the present study to evaluate the corrosion behavior of laser-alloyed layers. The values of corrosion potential (*E_corr_* = −187 mV) and corrosion current density (*I_corr_* = 31 × 10^−8^ A/cm^2^) of the surface layer, produced by LTPGN [9], were comparable to those-obtained in the case of the laser-alloyed layers in the present study. Although the value of *I_corr_* was slightly lower, the corrosion potential in 3.5% NaCl solution was higher only than that-obtained for the laser-alloyed layer with boron and Stellite-6. The high-temperature plasma gas nitrided layers [14], investigated in the same medium, were characterized by corrosion potentials from −325 to −190 mV depending on the nitriding duration. Such values were lower in comparison to the laser-alloyed layers with boron and selected metallic elements, except of the laser-alloyed layer with boron and Stellite-6. Simultaneously, the corrosion current densities were not calculated. Only passive current density was provided. Its values ranged from 10 × 10^−8^ to 20 × 10^−8^ A/cm^2^ in the case of the nitriding process, which was carried out for 30 min [14]. The HTPGN process resulted in the surface layers with corrosion potential from −599 to −518 depending on nitriding temperature [15]. These values were significantly more negative than those-reported for all the laser-alloyed layers in 3.5% NaCl solution. The corrosion current density was not provided. By contrast, the comparable values of *E_corr_* in 3.5% NaCl were obtained after CPEN process [16]. They ranged from −322 to 100 mV depending on the process parameters. The values of *Icorr* (from 9.2 × 10^−8^ to 81.5 × 10^−8^ A/cm^2^) also well corresponded to the corrosion current densities of the laser-alloyed layers, calculated in the present study (see Table 8). In the case of hybrid surface layers, produced by cold spraying (CS) of 316L steel followed by LTPGN, LTPGC, LTPGC and LTPGN as well as LTPGNC processes, the corrosion potentials in 3.5% NaCl ranged from −460 to −140 mV [21] and were usually slightly more negative than those-determined in the present work for laser-alloyed specimens, except of the laser-alloyed with boron and Stellite-6. Unfortunately, the values of *I_corr_* were not calculated [21]. The corrosion potential of low-temperature plasma gas carburized layer in 1 M H_2_SO_4_ solution was equal to approximately −80 mV and −200 mV without or with sliding, respectively [27]. Such values were slightly less negative than those-characteristic of the laser-alloyed layers in the present study. Simultaneously, the corrosion current density was not provided [27].

It was more difficult to compare the corrosion behavior of the laser-alloyed layers to other surface layers, produced by physical techniques, because of the differences in the media used. The potentiodynamic tests were also carried out in Ringer’s solution [2,6], aerated 3% NaCl [4,6,8], aerated 0.6 M NaCl [5], 0.5 M NaCl [7,10,27], 5% NaCl [17], natural seawater [24] or 0.5 M H_2_SO_4_ [19]. Usually, the determined or estimated values of corrosion potentials (*E_corr_*) were negative, obtaining the values from −50 mV (for low-temperature plasma gas nitrocarburized layer [7]) to −860 mV (for low-temperature gas nitrided layer with active screen [10]), except of the layers, produced by LTPGN, shot peening (SP) or hybrid treatment (SP followed by LTPGN) [17] which were examined in 5% NaCl solution. In these cases, the corrosion potential was positive, ranging from 135 to 875 mV [17]. 

The corrosion current density was rarely calculated [2,7,16,22,24]. The low-temperature plasma gas nitrided layer was characterized in Ringer;s solution by *I_corr_* value of 8 × 10^−8^ A/cm^2^ [2] or the comparable corrosion current density than that of the untreated 316L steel [6]. In the case of similar surface layers, tested in aerated 3% NaCl, the value of *I_corr_* was not provided [4] or was lower than that of 316L [6]. The corrosion current densities of low- and high-temperature plasma gas nitrided layers, examined in 0.6 M NaCl solution, were also given only descriptively [5]. It was indicated that *I_corr_* value, obtained after LTPGN process, was lower than that after HTPGN and comparable to that-characteristic of untreated 316L steel. The surface layers, fabricated by LTPGN and LTPGNC processes or by the same processes followed by producing the carbon coating (CC), were characterized by the relatively low values of *I_corr_* in 0.5 M NaCl [7]. They ranged from 1 × 10^−9^ to 7 × 10^−9^ A/cm^2^ [7]. The slightly higher corrosion current density was characteristic of carbon coated 316L steel 8 × 10^−9^ A/cm^2^ [7].

The values of *I_corr_* in the same solution after LTPGN processes with and without active screen (AS) were not calculated [10]. It was only reported that the corrosion current density after nitriding on the cathode was nearly the same to untreated austenitic steel, whilst after nitriding with active screen its value increased [10]. In the case of the 316L steel, subjected to LTPGN, SP followed by LTPGN or only shot-peened and tested in 5% NaCl solution [17], the values of *I_corr_* were not also calculated. It was only reported that current density often exceeded 1 × 10^−5^ A/cm^2^, except of the surface layer produced by SP followed by LTPGN at 370°C. The potentiodynamic tests of surface layers, produced in austenitic steel by LTPGC + LTPGN, SP + LTPGC + LTPGN or SP processes, were carried out in 0.5 M H_2_SO_4_ [19]. Although the corrosion current densities were not provided, the values of passive current density ranged from 4.55 × 10^−5^ to 1 × 10^−3^ A/cm^2^ or from 3.55 × 10^−5^ to 2.52 × 10^−4^ A/cm^2^ in the case of LTPGC followed by LTPGN or SP followed by LTPGC and LTPGN processes, respectively. Whereas the passive current density of shot-peened 316L steel was equal to 61.65 × 10^−5^ A/cm^2^. The LTPGN layers and the same layers with (Cr,W,Al,Ti,Si)N coating, examined in natural seawater, was characterized by the corrosion current densities of 5.38 × 10^−7^ and 1.29 × 10^−7^ A/cm^2^, respectively [24]. The values of *I_corr_* in 1 M H_2_SO_4_ and 0.5 M NaCl solutions were not calculated for low-temperature plasma gas carburized layer [27]. The similar layer was subjected to immersion test in 5.85% NaCl solution [26]. The evaluation of corrosion behavior of this layer consisted in the OM and SEM observations of the surface. The laser-alloyed layers with Cr and CrB_2_ were subjected to potentiodynamic tests in 3.6% HCl solution [62]. The corrosion potentials ranged from −449.3 to −405.8 mV, and the corrosion current densities were relatively high, obtaining values from 17030.5 × 10^−8^ to 36492.2 × 10^−8^ A/cm^2^.

The determined values of corrosion potential *E_corr_* and corrosion current density *I_corr_* of the surface layers, produced in the austenitic steel by the thermochemical techniques [35,36,41,44,52], are specified in Table 11. The relatively easy comparison with the laser-alloyed layers was possible only to the layers, produced by powder-pack boriding (P-PB), P-PB followed by P-PCr (powder-pack chromizing) or P-Cr followed by P-PB [41], LTGN [44] as well as LTC [52]. The corrosion behavior of these layers was studied in 3.5% NaCl solution, which was also used in the present study in relation to the laser-alloyed layers. Only the surface layer, fabricated by P-PB and subsequent P-PCr [41], obtained the comparable results with the laser-alloyed layers with boron or with boron and nickel. Although the corrosion potential was more negative (*E_corr_* = −216.7 mV), the corrosion current density (*I_corr_* = 24.64 × 10^−8^ A/cm^2^) was slightly lower than the values characteristic of the laser-alloyed layer mentioned above. The other layers, reported in the paper [41], were characterized by the worse corrosion behavior in 3.5% NaCl solution. The tribocorrosion tests of low-temperature gas nitrided layer in 3.5% NaCl resulted in the slightly more negative corrosion potentials (−90 mV without sliding and −300 mV with sliding) and considerably higher corrosion current densities (8.74 × 10^−4^ A/cm^2^ without sliding and 6.16 × 10^−3^ with sliding) [44] in comparison with the two indicated laser-alloyed layers of the most advantageous corrosion behavior. The corrosion potential of low-temperature carburized layer in 3.5% NaCl was equal to −10 mV, being comparable to the mentioned laser-alloyed layers. However, its corrosion current density was not calculated in this case.

The surface layers, produced by P-PB [35,36] or LTC [52] processes, were subjected to the potentiodynamic tests in other media, i.e., simulated body fluid (SBF) [35], 1 M HCl, 0.9% NaCl and 1 M NaOH [36] or 2.5 vol% HCl, 0.5 M H_2_SO_4_, 3 vol% CH_3_COOH and 2.5 vol% Oxonia [52]. Hence, it was difficult to compare their corrosion potentials and corrosion current densities to the values characteristic of the laser-alloyed layers.

The improvement in the corrosion behavior of all the considered surface layers has been usually ambiguous when compared to the untreated material (Table 9). Even if the values of their corrosion potentials and corrosion current densities were more advantageous or comparable, the untreated 316L steel was usually characterized by smaller region of active digestion and the wider region of primary passivation as well as the passive region. 

It is obvious that the potentiodynamic test is rather crude technique to analyze corrosion properties. It was commonly used in the analyzed works. However, without a doubt, electrochemical impedance spectroscopy (EIS) would be better technique, giving a possibility to model the circuit parameters [65]. Recently, EIS technique has been increasingly used to evaluate the corrosive properties of stainless steels [66,67,68,69]. There are also literature data indicating the use of this method for nitrided layers produced on stainless steels [24,70,71,72]. In the future, the corrosion behavior of laser-alloyed 316L steel should be analyzed using the electrochemical impedance spectroscopy. The use of the EIS technique would require the preparation of the new laser-alloyed samples according to the previously applied laser processing parameters.

## 4. Conclusions

The LSA of the austenitic 316L steel with boron and some metallic elements resulted in the formation of composite surface layers of advantageous functional properties. The effects of such a treatment on these properties allowed to formulate the following conclusions:

- A significant increase in hardness in the re-melted (i.e., laser-alloyed) zone was obtained in the case of all the variants of LSA. This hardness was several times greater than that of 316L steel without surface treatment,
- Although the hardness of laser-alloyed layers with boron as well as with boron and selected metallic elements was lower, their averaging depths were significantly higher than the depths of the surface layers produced on austenitic stainless steels using other physical and thermochemical techniques. These relatively high depths of the hardened surface layers could be important under conditions of appreciable mechanical wear;
- All the laser-alloyed layers were significantly more resistant to frictional wear compared to the untreated 316L steel, and their corrosion resistance did not diverge too much from the resistance of 316L steel;
- The proposed laser-alloyed layers were subjected to the wear tests at a relatively high load of 49 N and were characterized by the relatively significant increase in wear resistance, when compared to the majority of the other physical and thermochemical techniques of the surface treatment. The use of a relatively high load was possible due to the high depths of the laser-alloyed layers;
- In the case of other techniques of surface treatment, the greater effect on wear reduction was only obtained at much lower load or at the load which was difficult to determine. The higher load was reported rarely in literature data, and the improvement of wear resistance was not greater than that of laser-alloyed layers;
- The literature data indicated that the worn surface of the untreated austenitic 316L steel was characterized by obvious signs of strong plastic deformation as well as abrasive, adhesive and oxidative wear, as indicated by deep grooves, adhesive craters and presence of oxides;
- The wear behavior of all the laser-alloyed alloyed layers was characterized by a predominant abrasive wear as well as oxidative wear and to a lesser extent observed adhesive wear, which were confirmed by the shallow grooves, presence of oxides and adhesion craters, respectively;
- The wear mechanisms of the surface layers, fabricated by other physical and thermochemical techniques, were identified using various methods and devices. Hence, it was difficult to compare the wear mechanism of these layers to those-identified in laser-alloyed layers. However, many of these surface layers were also characterized by abrasive, adhesive as well as the oxidative wear. Sometimes, the severe plastic deformations of the substrate material underneath the surface layer were observed;
- Despite the composite and multiphase microstructure of laser-alloyed layers (iron, nickel and chromium borides in the austenitic matrix), their corrosion resistance did not diverge too much from the resistance of 316L steel without surface treatment;
- From an application point of view, the LSA with boron and nickel seemed to be the most advantageous variant of the treatment. The laser-alloyed layer with very-high wear resistance had the best corrosion resistance (measured by corrosion potential and corrosion current density), similar to the untreated 316L steel in both corrosive media analyzed (1 M H_2_SO_4_ and 3.5% NaCl);
- The effect of the other surface layers (produced by physical or thermochemical techniques) on the corrosion resistance could be compared to the corrosion behavior of laser-alloyed layers only, if the same corrosive media were used during potentiodynamic tests. Only some of these layers were characterized by comparable values of corrosion potential and corrosion current density to laser-alloyed layers;
- The improvement in the corrosion behavior of all the considered surface layers has been usually ambiguous when compared to the untreated austenitic steel. Even if their corrosion potentials were higher and corrosion current densities were lower or comparable, the untreated 316L steel was usually characterized by smaller region of active digestion and the wider region of primary passivation as well as the wider passive region;
- The usefulness of the proposed laser surface alloying to increase hardness and wear resistance of 316L steel without sacrificing its corrosion resistance was confirmed.

## Figures and Tables

**Table 1:** Maximal hardness values and averaging depths of the surface layers measured after the surface treatment of austenitic steel using various physical techniques.

Type of Surface Treatment	Maximal Hardness of Surface Layer (HV, HK, GPa, HV_IT_)	Averaging Depth of Surface Layer (μm)	Reference
LTPGN	1200 HV0.05	4	[2]
LTPGN	1080 HV0.05	4	[3]
LTPGN	572 HV0.2	4.07	[5]
LTPGN	1218 HV0.05	9	[7]
LTPGN	2175 HV0.0036	6.5	[8]
LTPGN	1194–1454 HV0.01	13–16	[9]
LTPGN	720 HK0.01	2	[10]
LTPGN with active screen	1100 HK0.01	6	[10]
LTPGNC	962 HV0.05	10.5	[7]
HTPGN	1180 HV0.05	20	[3]
HTPGN	1196 HV0.2	41.6	[5]
HTPGN	1340 HV0.05	12	[14]
HTPGN	1060 HV	31.31	[15]
CPEN	438 HV0.1	28.06	[16]
SP + LTPGN	1615–1662 HV0.005	4.2–5.7	[17]
LTPGC + LTPGN	5–9 GPa	55–80	[19]
SP	6 GPa	70–80	[19]
SP + LTPGC + LTPGN	7.5–11.5 GPa	65–90	[19]
SLM + LTPGN	675 HV0.01	18	[20]
CS of 316L + LTPGN	950–1350 HV0.025	3.8–12.5	[21]
CS of 316L + LTPGC	800–1000 HV0.025	6.5–13.7	[21]
CS of 316L + LTPGC + LTPGN	1250–1300 HV0.025	7–16.7	[21]
CS of 316L + LTPGNC	1100–1350 HV0.025	6.9–17.2	[21]
LMD of 316L and Ni + LTPGN	1103–1288 HV0.025	8.2–9.7	[22]
LTPGN	1120 HV0.05	15	[23]
LTPGN + MAIP	2280 HV0.05	2.4 + 15	[23]
LTPGC	11–11.8 GPa,	14	[26]
LTPGC	900 HV0.025	25	[27]
LTPGC	570–930 HV0.025	7.5–21	[28]
PPB	28.093 GPa	7.6	[31]
TiN coating produced by PVD	18.7 GPa	1.4	[53]
TiN coating produced by PVD	26 GPa	1.6–2.4	[54]
LSA with Cr_3_C_2_	480 HV	a few micrometers	[57]
LSA with NiCoCrB	410 HV0.2	260–740	[59]
LSA with B, *P* = 1.82 kW, *DR* = 0.37	796 HV0.1	365	[60]
LSA with B, *P* = 1.82 kW, *DR* = 0.54	595 HV0.1	432 *	this work and [63] *
LSA with B + Stellite-6, *P* = 1.43 kW	841 HV0.1	338 *	this work and [63] *
LSA with B + Stellite-6, *P* = 1.56 kW	771 HV0.1	384 *	this work and [63] *
LSA with B + Ni, *P* = 1.43 kW	724 HV0.1	345 *	this work and [63] *
LSA with B + Ni, *P* = 1.56 kW	675 HV0.1	383 *	this work and [63] *
LSA with B + Ni-Cr, *P* = 1.43 kW	911 HV0.1	352 *	this work and [63] *
LSA with B + Ni-Cr, *P* = 1.56 kW	841 HV0.1	395 *	this work and [63] *

* Averaging depth of laser re-melted zone (laser-alloyed layer).

**Table 2:** Maximal hardness values and averaging depths of the surface layers measured after the surface treatment of austenitic steel using various thermochemical techniques.

Type of Surface Treatment	Maximal Hardness of Surface Layer (HV, HK, GPa, HV_IT_)	Averaging Depth of Surface Layer (μm)	Reference
P-PB	1800 HV	7–87	[33]
Surface mechanical attrition treatment (SMAT) + P-PB	2000 HV0.05	11–35	[34]
P-PB	1836–2227 HK0.05	2.3–25	[35]
P-PB	18 GPa	15–24	[37]
P-PB + diffusion annealing	15 GPa	15–23	[37]
P-PB	19–21 GPa	56.82	[38]
P-PB	24 GPa	28.18	[39]
P-PB	2000 HV_IT_	10.21–51.25	[40]
P-PB	1580 HV0.05	46	[41]
P-PB + P-PCr	1800 HV0.05	45	[41]
P-PCr + P-PB	1610 HV0.05	55	[41]
Boriding in liquid medium	22 GPa	5–40	[42]
LTGN	1300 HV0.05	6	[44]
LTGN of HVOF-sprayed 316L steel	874–1005 HV0.001	difficult to measure	[45]
Nitriding in liquid medium	11.5 GPa	1.8–7.2	[49]
LTC	1100 HV0.05	30	[52]

**Table 3:** Effect on wear in comparison with untreated austenitic steel using the various methods of wear tests and wear evaluations after the surface treatment by various physical techniques.

Type of Surface Treatment	Wear Test Technique/Load (N)	Method of Wear Evaluation	Effect on Wear Compared to Untreated Austenitic Steel	Reference
LTPGN	block-on-ring/?	volumetric wear	347- or 371-times lower	[2,3]
LTPGN	ball-on-disc/8.3	volumetric wear	40-times lower	[5]
LTPGN	ball-on-disc/10	specific wear rate coefficient of friction	29-times lower 1.015-times higher	[7]
LTPGN	pin-on-disc/100	mass loss coefficient of friction	4.6–91-times lower 1.075–1.47-times higher	[8]
LTPGN	ball-on-disc/20	volumetric wear coefficient of friction	13.5-times lower 1.026-times lower	[9]
LTPGNC	ball-on-disc/10	specific wear rate coefficient of friction	23-times lower 1.063-times lower	[7]
HTPGN	block-on-ring/?	volumetric wear	920-times lower	[3]
HTPGN	ball-on-disc/8.3	volumetric wear	2.5-times lower	[5]
HTPGN	ball-on-disc/3	specific wear rate	175–650-times lower	[15]
CPEN	ball-on-disc/3	coefficient of friction	1.6-times lower	[16]
SP + LTPGC + LTPGN	ball-on-disc/15	volumetric wear	up to 65-times lower	[19]
CS of 316L + LTPGN	ball-on-disc/1.96	specific wear rate	10–26-times lower	[21]
CS of 316L + LTPGC	ball-on-disc/1.96	specific wear rate	4–5-times lower	[21]
CS of 316L + LTPGC + + LTPGN	ball-on-disc/1.96	specific wear rate	9–26-times lower	[21]
CS of 316L + LTPGNC	ball-on-disc/1.96	specific wear rate	4–16-times lower	[21]
LMD of 316L and Ni + LTPGN	ball-on-disc/1.96	specific wear rate	56–175-times lower	[22]
LTPGN	ball-on-disc/10	volumetric wear coefficient of friction	1.47-times lower 1.26-times lower	[23]
LTPGN + MAIP	ball-on-disc/10	volumetric wear coefficient of friction	11.2-times lower 2.25-times lower	[23]
LTPGC	ball-on-disc/10	% of volume removed	1.67–14.29-times lower	[26]
LTPGC	ball-on-disc/20	volumetric wear	10-times lower	[27]
PPB	block-on-ring/19.6	mass wear int. factor relative mass loss	4-times lower 6-times lower	[31]
TiN coating produced by PVD	ball-on-disc/5	mass loss/distance coefficient of friction	2.52-times lower 1.37-times lower	[53]
TiN coating produced by PVD	ball-on-disc/5	specific wear rate coefficient of friction	up to 13.67-times lower 1.11–5-times lower	[54]
LSA with Cr_3_C_2_ + Cr	pin-on-disc/5	specific wear rate coefficient of friction	1.29-times lower no change in the value	[57]
LSA with B, *P* = 1.82 kW, *DR* = 0.37	block-on-ring/49	mass wear int. factor relative mass loss	15-times lower 5-times lower	[60]
LSA with B, *P* = 1.82 kW, *DR* = 0.54	block-on-ring/49	mass wear int. factor relative mass loss	20-times lower 19-times lower	this work
LSA with B + Stellite-6, *P* = 1.43 kW	block-on-ring/49	mass wear int. factor relative mass loss	26-times lower 21-times lower	this work
LSA with B + Stellite-6, *P* = 1.56 kW	block-on-ring/49	mass wear int. factor relative mass loss	20-times lower 21-times lower	this work
LSA with B + Ni, *P* = 1.43 kW	block-on-ring/49	mass wear int. factor relative mass loss	25-times lower 26-times lower	this work
LSA with B + Ni, *P* = 1.56 kW	block-on-ring/49	mass wear int. factor relative mass loss	22-times lower 19-times lower	this work
LSA with B + Ni-Cr, *P* = 1.43 kW	block-on-ring/49	mass wear int. factor relative mass loss	24-times lower 26-times lower	this work
LSA with B + Ni-Cr, *P* = 1.56 kW	block-on-ring/49	mass wear int. factor relative mass loss	16-times lower 16-times lower	this work

“?” – the load was unknown.

**Table 4:** Effect on wear in comparison with untreated austenitic steel using the various methods of wear tests and wear evaluations after the surface treatment by various thermochemical techniques.

Type of Surface Treatment	Wear Test Technique/Load (N)	Method of Wear Evaluation	Effect on Wear Compared to Untreated Austenitic Steel	Reference
P-PB	ball-on-disc/5	specific wear rate coefficient of friction	4–9-times lower 1.31–4.13-times lower	[35]
P-PB	ball-on-disc/5 or 20	specific wear rate coefficient of friction	38–62-times lower unknown	[38]
P-PB	ball-on-disc/0.2 in abrasive slurry	specific wear rate	1.53-times lower	[39]
P-PB	ball-on-disc/5	volumetric wear specific wear rate coefficient of friction	unknown 15–59-times lower 2.03–2.68-times lower	[40]
P-PB	pin-on-disc/95	mass loss coefficient of friction	4.75-times lower 1.055-times higher	[41]
	pin-on-disc/125	mass loss coefficient of friction	3-times lower 1.15-times higher	
P-PB + P-PCr	pin-on-disc/95	mass loss coefficient of friction	11-times lower 1.17-times higher	[41]
	pin-on-disc/125	mass loss coefficient of friction	3.71-times lower 1.08-times lower	
P-PCr + P-PB	pin-on-disc/95	mass loss coefficient of friction	2-times lower 1.22-times higher	[41]
	pin-on-disc/125	mass loss coefficient of friction	1.81-times lower no effect	
LTGN	ball-on-disc/2 (tribocorrosion test in 3.5% NaCl)	mass loss	5–70-times lower	[44]
	ball-on-disc/10 (tribocorrosion test in 3.5% NaCl)	mass loss coefficient of friction	1.56-times higher 1.11-times lower	
LTGN of HVOF-sprayed 316L steel	ball-on-disc/20	wear area wear depth	2.07–9-times lower 1.74–5.53-times lower	[45]
LTGN of HVOF-sprayed 316L steel	reciprocating ball-on-plane/26	volumetric wear wear depth	1.75–2.92-times lower 1.42–2.16-times lower	[45]
LTC	block-on-ring/5–25	volumetric wear	up to 2-times lower	[52]

**Table 5:** Wear mechanisms of the surface layers produced by various physical techniques.

Type of Surface Treatment	Method of Wear Mechanism Identification	Signs of Wear Mechanism on the Worn Surface	Wear Mechanism	Reference
LTPGN	OM images	craters with scratching and rolling	grooving abrasion rolling abrasion	[5]
LTPGN	OM images optical profilometer	shallow grooves adhesion craters transverse cracks	abrasive wear adhesive wear plastic deformation of the material underneath the surface layer	[7]
LTPGN	OM images	shallow grooves	abrasive wear	[9]
LTPGNC	OM images optical profilometer	shallow grooves adhesion craters transverse cracks	abrasive wear adhesive wear plastic deformation of the material underneath the surface layer	[7]
HTPGN	OM images	craters with scratching and rolling	grooving abrasion rolling abrasion	[5]
HTPGN	SEM images WDS patterns	shallow grooves increased oxygen content (presence of oxides)	abrasive wear oxidative wear	[15]
CPEN	confocal microscope	shallow grooves	abrasive wear	[16]
LTPGN	SEM images EDS analysis	slight scratches craters (dark pitches) increased oxygen content (presence of oxides)	abrasive wear adhesive wear oxidative wear	[23]
LTPGN + MAIP	SEM images EDS analysis	slight scratches increased oxygen content (presence of oxides)	abrasive wear oxidative wear	[23]
LTPGC	OM images	shallow grooves signs of general corrosion (oxygen evolution) in 1 M H_2_SO_4_ signs of crevice corrosion in 0.5 M NaCl	abrasive wear corrosive wear corrosive wear	[27]
TiN coating produced by PVD	SEM images	scratches	abrasive wear	[53]
TiN coating produced by PVD	SEM images EDS analysis	plough action pitting and delamination increased oxygen content (presence of oxides)	abrasive wear adhesive wear oxidative wear	[54]
LSA with B, *P* = 1.82 kW, *DR* = 0.37	SEM images EDS patterns	shallow grooves adhesion craters increased oxygen content (presence of oxides)	abrasive wear adhesive wear oxidative wear	[60]
all the LSA processes with B, B + Stellite-6, B + Ni or B + Ni-Cr	SEM images EDS patterns	shallow grooves adhesion craters increased oxygen content (presence of oxides)	abrasive wear adhesive wear oxidative wear	this work

**Table 6:** Wear mechanisms of the surface layers produced by various thermochemical techniques.

Type of Surface Treatment	Method of Wear Mechanism Identification	Signs of Wear Mechanism on the Worn Surface	Wear Mechanism	Reference
P-PB	SEM images EDS analysis	deep scars (scratching) adhesion craters increased oxygen content (presence of oxides)	abrasive wear adhesive wear oxidative wear (predominant in SBF medium)	[35]
P-PB	optical profilometer and literature data [64]	grooves and material shedding (debris) intergranular cracks surface cracks not indicated	abrasive wear pitting cracking oxidative wear	[38]
P-PB	optical profilometer and analysis of percentage of SiC particles in the slurry	high percentage of abrasive SiC particles in the slurry and low load low percentage of abrasive SiC particles in the slurry and high load	rolling abrasion grooving abrasion	[39]
P-PB	SEM images	micro-cracking micro-cutting and micro-plowing micro-fatigue debris-adhesion and delaminations not indicated	suffer fracture abrasive wear pitting adhesive wear plastic deformations	[40]
LTGN	SEM images (tribocorrosion test in 3.5% NaCl)	abrasion marks micro-pits	abrasive wear corrosive wear	[44]
LTGN of HVOF-sprayed 316L steel	SEM images	deep grooves and breakout of hardened spray particles	abrasive wear	[45]

**Table 7:** Corrosion potential *E_corr_* and corrosion current density *I_corr_* of laser-alloyed layers, produced in 316L austenitic steel, compared to the same material without surface treatment based on the polarization curves in 1 M H_2_SO_4_ solution.

Type of Surface Treatment	Corrosion Potential *E_corr_* (mV)	Corrosion Current Density *I_corr_* (A/cm^2^)
316L steel without surface treatment	−304.11	10.1 × 10^−6^
Laser-alloyed 316L steel with B (*DR* = 0.54)	−279.59	9 × 10^−6^
Laser-alloyed 316L steel with B and Stellite-6	−356.88	15 × 10^−6^
Laser-alloyed 316L steel with B and Ni	−283.35	4.5 × 10^−6^
Laser-alloyed 316L steel with B and Ni-Cr	−286.89	7.4 × 10^−6^

**Table 8:** Corrosion potential *E_corr_* and corrosion current density *I_corr_* of laser-alloyed layers, produced in 316L austenitic steel, compared to the same material without surface treatment based on the polarization curves in 3.5% NaCl solution.

Type of Surface Treatment	Corrosion Potential *E_corr_* (mV)	Corrosion Current Density *I_corr_* (A/cm^2^)
316L steel without surface treatment	9.87	52.2 × 10^−8^
Laser-alloyed 316L steel with B (*DR* = 0.54)	−10.97	63.8 × 10^−8^
Laser-alloyed 316L steel with B and Stellite-6	−371.19	64.6 × 10^−8^
Laser-alloyed 316L steel with B and Ni	43.32	99.7 × 10^−8^
Laser-alloyed 316L steel with B and Ni-Cr	−132.84	22.5 × 10^−7^

**Table 9:** Corrosion potential *E_corr_* and corrosion current density *I_corr_* of the untreated, as-sprayed and as-deposited austenitic 316L steels based on the other papers.

Type of Investigated Material	Medium Used	Corrosion Potential *E_corr_* (mV)	Corrosion Current Density *I_corr_* (A/cm^2^)	Reference
untreated 316L	Ringer’s solution	−162	1.5 × 10^−8^	[2]
untreated 316L	aerated 3% NaCl	−395 *	not calculated	[4]
untreated 316L	aerated 0.6 M NaCl	−152	not calculated	[5]
untreated 316L	Ringer’s solution aerated 3% NaCl	15 * 50	not calculated 6.3 × 10^−5^	[6]
untreated 316L	0.5 M NaCl	−140	8 × 10^−9^	[7]
untreated 316L	aerated 3% NaCl	−400	not calculated	[8]
untreated 316L	3.5% NaCl	−241	55 × 10^−8^	[9]
untreated 316L	0.5 M NaCl	−430 *	not calculated	[10]
untreated 316L	3.5% NaCl	−275 *	not calculated	[14]
untreated 316L	3.5% NaCl	−946	not calculated	[15]
untreated 316L	3.5% NaCl	−290	54.7 × 10^−8^	[16]
untreated 316L	5% NaCl	410 *	not calculated	[17]
untreated 316L	0.5 M H_2_SO_4_	−373	not calculated	
as-sprayed 316L (by CS)	3.5% NaCl	−270 *	not calculated	[21]
as-deposited 316L (by LMD)	3.5% NaCl	−249	1.0 × 10^−3^	[22]
untreated 316L	natural seawater	−420	4.94 × 10^−6^	[24]
untreated 316L	1 M H_2_SO_4_ (tribocorrosion test) 0.5 M NaCl (tribocorrosion test)	−210 without sliding * −280 with sliding * −180 without sliding * −440 with sliding *	not calculated not calculated not calculated not calculated	[27]
untreated 316L	SBF	from −266 to −255	from 5.8 × 10^−8^ to 7.4 × 10^−8^	[35]
untreated 316L	1 M HCl 0.9% NaCl 1 M NaOH	from −404 to −356 from −204 to −186 from −321 to −297	from 3141.3 × 10^−8^ to 4014.7 × 10^−8^ from 13.1 × 10^−8^ to 16.1 × 10^−8^ from 12 × 10^−8^ to 78.4 × 10^−8^	[36]
untreated 316L	3.5% NaCl	−163.1	52.68 × 10^−8^	[41]
untreated 316L	3.5% NaCl (tribocorrosion test)	−160 without sliding * −360 with sliding *	1.42 × 10^−3^ without sliding 1.66 × 10^−1^ with sliding	[44]
untreated 316L	3.5% NaCl 2.5 vol% HCl 0.5 M H_2_SO_4_ 3 vol% CH_3_COOH 2.5 vol% Oxonia	−150 −400 −360 60 390	not calculated not calculated not calculated not calculated not calculated	[52]

* the values estimated based on the polarization curves.

**Table 10:** Corrosion potential *E_corr_* and corrosion current density *I_corr_* of surface layers, produced in 316L austenitic steel using other physical techniques.

Type of Surface Treatment	Medium Used	Corrosion Potential *E_corr_* (mV)	Corrosion Current Density *I_corr_* (A/cm^2^)	Reference
LTPGN	Ringer’s solution	−179	8 × 10^−8^	[2]
LTPGN	aerated 3% NaCl	from −160 to −110	not calculated	[4]
LTPGN	aerated 0.6 M NaCl	−53	lower than after HTPGN and comparable to 316L	[5]
HTPGN	aerated 0.6 M NaCl	−575	greater than after LTPGN	[5]
LTPGN	Ringer’s solution aerated 3% NaCl	from −330 to −240 * from −260 to −110 *	comparable to 316L steel lower than that of 316L	[6]
LTPGN	0.5 M NaCl	−150	7 × 10^−9^	[7]
LTPGNC	0.5 M NaCl	−50	3 × 10^−9^	[7]
LTPGN + CC	0.5 M NaCl	−77	4 × 10^−9^	[7]
LTPGNC + CC	0.5 M NaCl	−77	1 × 10^−9^	[7]
CC 316L	0.5 M NaCl	−194	8 × 10^−9^	[7]
LTPGN	aerated 3% NaCl	from −200 to −120	not calculated	[8]
LTPGN	3.5% NaCl	−187	31 × 10^−8^	[9]
LTPGN	0.5 M NaCl	−600 *	not calculated	[10]
LTPGN with AS	0.5 M NaCl	−870 *	not calculated	[10]
HTPGN	3.5% NaCl	from −325 to −190 *	not calculated	[14]
HTPGN	3.5% NaCl	from −599 to −518	not calculated	[15]
CPEN	3.5% NaCl	from −322 to +100	from 9.2 × 10^−8^ to 81.5 × 10^−8^	[16]
LTPGN	5% NaCl	330−875 *	not calculated	[17]
SP + LTPGN	5% NaCl	135−425 *	not calculated	[17]
SP 316L	5% NaCl	350 *	not calculated	[17]
LTPGC + LTPGN	0.5 M H_2_SO_4_	from −359 to −275	not calculated	[19]
SP + LTPGC + LTPGN	0.5 M H_2_SO_4_	from −351 to −301	not calculated	[19]
SP 316L	0.5 M H_2_SO_4_	−356	not calculated	[19]
CS of 316L + LTPGN	3.5% NaCl	from −255 to −220 *	not calculated	[21]
CS of 316L + LTPGC	3.5% NaCl	from −215 to −140 *	not calculated	[21]
CS of 316L + LTPGC + LTPGN	3.5% NaCl	from −290 to −180 *	not calculated	[21]
CS of 316L + LTPGNC	3.5% NaCl	from −460 to −170 *	not calculated	[21]
LMD of 316L and Ni + LTPGN	3.5% NaCl	from −405 to −260	from 2.7 × 10^−4^ to 1.1 × 10^−3^	[22]
LTPGN	natural seawater	−220	5.38 × 10^−7^	[24]
LTPGN + (Cr,W,Al,Ti,Si)N coating produced by PVD	natural seawater	−310	1.29 × 10^−7^	[24]
LTPGC	1 M H_2_SO_4_ 0.5 M NaCl	−80 without sliding * −200 with sliding * −200 without sliding * −440 with sliding *	not calculated not calculated not calculated not calculated	[27]
LSA with Cr + CrB_2_	3.6% HCl	from −449.3 to −405.8	from 17030.5 × 10^−8^ to 36492.2 × 10^−8^	[62]

* The values estimated based on the polarization curves.

**Table 11:** Corrosion potential *E_corr_* and corrosion current density *I_corr_* of surface layers, produced in 316L austenitic steel using thermochemical techniques.

Type of Surface Treatment	Medium Used	Corrosion Potential *E_corr_* (mV)	Corrosion Current Density *I_corr_* (A/cm^2^)	Reference
P-PB	SBF	from −574 to −386	from 90.8 × 10^−8^ to 1479 × 10^−8^	[35]
P-PB	1 M HCl 0.9% NaCl 1 M NaOH	from −357 to −244 from −598 to −345 from −457 to −273	from 620.9 × 10^−8^ to 3224 × 10^−8^ from 71.7 × 10^−8^ to 3829.6 × 10^−8^ from 17.9 × 10^−8^ to 1932 × 10^−8^	[36]
P-PB	3.5% NaCl	−590.7	159.9 × 10^−8^	[41]
P-PB + P-PCr	3.5% NaCl	−216.7	24.64 × 10^−8^	
P-PCr + P-PB	3.5% NaCl	−357.4	136.6 × 10^−8^	
LTGN	3.5% NaCl (tribocorrosion test)	−90 without sliding * −300 with sliding *	8.74 × 10^−4^ without sliding 6.16 × 10^−3^ with sliding	[44]
LTC	3.5% NaCl 2.5 vol% HCl 0.5 M H_2_SO_4_ 3 vol% CH_3_COOH 2.5 vol% Oxonia	−10 −230 70 70 560	not calculated not calculated not calculated not calculated not calculated	[52]

* The values estimated based on the polarization curves.

## Data Availability

The authors confirm that the data supporting the findings of this study is available within the article.
